# Single-layered organic photovoltaics with double cascading charge transport pathways: 18% efficiencies

**DOI:** 10.1038/s41467-020-20580-8

**Published:** 2021-01-12

**Authors:** Ming Zhang, Lei Zhu, Guanqing Zhou, Tianyu Hao, Chaoqun Qiu, Zhe Zhao, Qin Hu, Bryon W. Larson, Haiming Zhu, Zaifei Ma, Zheng Tang, Wei Feng, Yongming Zhang, Thomas P. Russell, Feng Liu

**Affiliations:** 1 Frontiers Science Center for Transformative Molecules, In-situ Center for Physical Science, and Center of Hydrogen Science, School of Chemistry and Chemical Engineering, Shanghai Jiao Tong University, Shanghai, 200240 People’s Republic of China; 2 Department of Polymer Science and Engineering, University of Massachusetts, Amherst, MA 01003 USA; 3 Chemistry & Nanoscience Department, National Renewable Energy Laboratory, Golden, Colorado 80401 USA; 4 Department of Chemistry, Zhejiang University, Hangzhou, 310027 People’s Republic of China; 5 Center for Advanced Low-dimension Materials, State Key Laboratory for Modification of Chemical Fibers and Polymer Materials, College of Materials Science and Engineering, Donghua University, Shanghai, 201620 People’s Republic of China; 6 State Key Laboratory of Fluorinated Functional Membrane Materials and Dongyue Future Hydrogen Energy Materials Company, Zibo City, 256401 Shandong Province People’s Republic of China

**Keywords:** Energy, Electronic devices, Polymers

## Abstract

The chemical structure of donors and acceptors limit the power conversion efficiencies achievable with active layers of binary donor-acceptor mixtures. Here, using quaternary blends, double cascading energy level alignment in bulk heterojunction organic photovoltaic active layers are realized, enabling efficient carrier splitting and transport. Numerous avenues to optimize light absorption, carrier transport, and charge-transfer state energy levels are opened by the chemical constitution of the components. Record-breaking PCEs of 18.07% are achieved where, by electronic structure and morphology optimization, simultaneous improvements of the open-circuit voltage, short-circuit current and fill factor occur. The donor and acceptor chemical structures afford control over electronic structure and charge-transfer state energy levels, enabling manipulation of hole-transfer rates, carrier transport, and non-radiative recombination losses.

## Abstract (web summary)

Efficiency of organic solar cells is determined by the physical properties of donors and acceptors in bulk heterojunction film. The authors optimise quaternary blends to realize a double cascading energy level alignment enabling efficient carrier dissociation and transport, achieving 18% efficiency.

## Introduction

A holistic strategy that can simultaneously improve open-circuit voltage (*V*_OC_)^1–3^, short-circuit current (*J*_SC_)^4,5^, and fill factor (FF)^6–9^ has been long sought in organic photovoltaics (OPVs) to maximize power conversion efficiency (PCE). Improving all characteristics simultaneously has been hard to achieve, due to morphological and electronic structure constraints, leading to performance trade-offs. The ideal OPV device simultaneously maximizes light absorption, enhances exciton splitting, and facilitates the carrier extraction. The carrier generation kinetics must shunt energy loss channels induced by low energy charge-transfer (CT) states to improve the *V*_OC_^10,11^. Consequently, optimizing the electronic structure of photovoltaic blends along with optimizing morphology is essential^12–14^. Mixtures of the mid-bandgap donor PM6 and the low-bandgap acceptor Y6 have shown exceptional PCE (~16%), high *J*_SC_ (~25 mA cm^−2^), and low energy loss (*E*_loss_, 0.5–0.6 eV)^15–19^. Enhancement of the photovoltaic characteristics requires more efficient exciton splitting and carrier transport pathways in the active layer.

Here, we use quaternary blends, comprised of existing donor–acceptor pairs mixed with additional donor and acceptor components to mediate deficiencies in electronic performance or morphology, to address this challenge. It is advantageous to establish a tiered energy level alignment, to form cascading charge hopping channels that mitigate the *J*_SC_ loss by fine-tuning the charge splitting and allow manipulation of multiple charge-transfer energies to ensure a high *V*_OC_. This applies to both electron and hole transport pathways to maximize the energy gain. From the hole transport side, PM7, which has a chemical structure similar to PM6 but a deeper highest occupied molecular orbital (HOMO) energy level, can be used as an “added” donor^20,21^, since it is miscible with PM6, does not disrupt the morphology, and provides a tiered cascading energy level alignment. From the electron transport side, PC_71_BM, which is compatible with the base PM6:Y6 blends, is an acceptor with a slightly higher lowest unoccupied molecular orbital (LUMO) than Y6 that affords a cascading energy level alignment to improve electron transport and promote efficiency. Consequently, the quaternary blend strategy, where the composition can be fine-tuned to optimize device characteristics, represents a new strategy to improve PCEs.

In addition to reducing the charge splitting driving force, PM7 preferentially interacts with the Y6 acceptor, effectively regulating the crystallization of Y6 to better suit carrier transport. PM7 redirects the ultrafast hole transfer from Y6 to the donor phase, forming tiered cascading energy levels that improve *J*_SC_ without further absorption gain. Adding PC_71_BM reduces the light absorption, yet not the current output. The higher LUMO level of PC_71_BM in the acceptor mixture improves the *V*_OC_ and electron transport, and offsets the absorption loss, leading to an enhancement in device performance. The synergy between the components in the PM6:PM7:Y6:PC_71_BM quaternary devices result in a maximum PCE of 18.07%, the highest in single-layered OPV devices, with an excellent stability (81% PCE after 1000 h illumination) that is ~5% better than PM6:Y6 binary devices. The double cascading quaternary blend strategy is implemented in other material systems, which is reflected in similar behavior of device operation, demonstrating the novelty in the approach for OPV device fabrication. These results demonstrate the importance of manipulating the electronic structure in BHJ thin films, while simultaneously manipulating the morphology, opening a new route to higher efficiency OPV devices.

## Results

### The double cascading charge transport and device performance

**Fig. 1:**
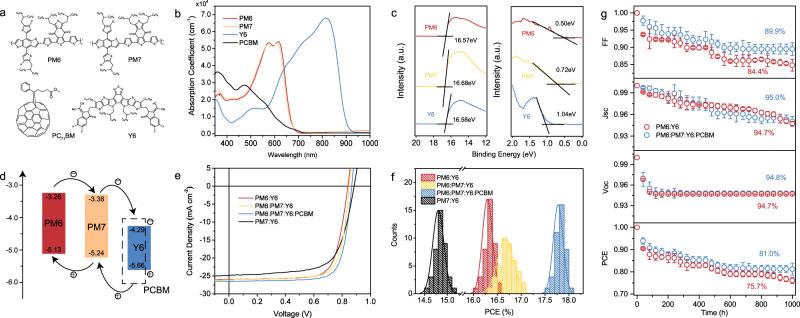
Molecular structure and photovoltaic performance of single-junction devices. **a** Chemical structures, **b** thin film absorption coefficients, and **c** UPS results of PM6 (red line), PM7 (yellow line), and Y6 (blue line). Fermi energy was determined by linear extrapolating the high binding energy portion of the spectrum, and HOMO energy level was referred to low binding energy onset. **d** Energy level alignment and double cascading transport pathways for quaternary system. **e** Current density–voltage characteristics and **f** histogram of PCE measurement for 40 devices on binary, ternary, and quaternary devices under constant incident light intensity (AM 1.5 G, 100 mW cm^−2^). **g** Normalized PCE, *V*_OC_, *J*_SC_, and FF (20 devices statistics) against aging time under illumination equivalent to ~1 sun for 1000 h (red for binary and blue for quaternary, the error bar is from the deviation of twenty samples).

Figure 1a, b shows the chemical structure and absorption profiles of the materials used in this study. The Y6 acceptor absorbs from 700 to 950 nm, while PM6 and PM7 donors show complementary absorption from 400 to 700 nm. PC_71_BM, with a much lower absorption in VIS-IR region, can mostly be considered a transport medium. The HOMO and LUMO are −5.13 eV/−3.28 eV for PM6, −5.24 eV/−3.38 eV for PM7, −5.66 eV/−4.29 eV for Y6, and −6.10 eV/−4.10 eV for PC_71_BM. The HOMO levels are accurately measured by ultraviolet photoelectron spectroscopy (UPS)^22^, coupled with the optical band gaps to estimate the LUMO levels (Fig. 1c, d). Solar cells were prepared with a forward structure (ITO/PEDOT:PSS/active layer/PFNDI-Br/Ag). The donor to acceptor ratio was fixed at 1:1.2 (optimized conditions for PM6:Y6) to avoid light-absorption-induced performance change, and PC_71_BM was added separately. The concentrations of PM7 and PC_71_BM were varied to determine the optimal composition. Detailed performances of the devices are shown in Supplementary Fig. 1, and Supplementary Tables 1 and 2. For the PM6:PM7:Y6 ternary blends, a mixing ratio of 0.8:0.2:1.2 (*D*_1_:*D*_2_:*A*_1_) was found to be optimal, while for the PM6:PM7:Y6:PC_71_BM quaternary blends, a mixing ratio of 0.8:0.2:1.2:0.25 (*D*_1_:*D*_2_:*A*_1_:*A*_2_) yielded optimum performance. The current density–voltage (*J–V*) curves are shown in Fig. 1e, with performance detailed in Supplementary Fig. 2 and Table 1. PM6:Y6 binary devices showed a maximum PCE of 16.52%, with a *V*_OC_ of 0.842 V, a *J*_SC_ of 25.98 mA cm^−2^, and an FF of 75.52%. Ternary devices showed a maximum PCE of 17.02%, a *V*_OC_ of 0.848 V, a *J*_SC_ of 26.17 mA cm^−2^, and an FF of 76.70%. Quaternary devices showed a maximum PCE of 18.07%, with a *V*_OC_ of 0.859 V, a *J*_SC_ of 26.55 mA cm^−2^, and an FF of 79.23%. The quaternary devices had a certified PCE of 17.35%, subject to the calibration procedures of the National Renewable Energy Laboratory, using a 0.032 cm^2^ photon mask (Supplementary Fig. 3), which is the highest certified value reported for a single-layered BHJ device. In the ternary blends, the external quantum efficiency (EQE) improved slightly at ~640 nm in comparison to the PM6:Y6 binary blends, as shown in Supplementary Fig. 4. In the quaternary blends, the EQE spectra of 450–600 nm and 650–800 nm improved slightly. Therefore, enhanced electron transport aided in improving the light extraction from the acceptor materials. The enhanced performance results from better charge collection channels for both electrons and holes, i.e., double cascading carrier transport pathways, which will be discussed in detail in the following section.

**Table 1:** Photovoltaics of BHJ solar cells under illumination of AM 1.5 G, 100 mW cm^−2^.

Blend	*V*_OC_ (V)	*J*_SC_ (mA cm^−2^)	FF (%)	PCE (%)	*μ*_e_ (cm^2^ s^−1^ V^−1^)	*μ*_h_ (cm^2^ s^−1^ V^−1^)
PM6:Y6	0.842 (0.842 ± 0.001)	25.98 (25.67 ± 0.19)	75.52 (74.91 ± 0.66)	16.52 (16.30 ± 0.11)	(4.42 ± 0.36) × 10^−4^	(1.41 ± 0.49) × 10^−3^
PM6:PM7:Y6	0.848 (0.847 ± 0.001)	26.17 (25.72 ± 0.31)	76.70 (75.91 ± 0.72)	17.02 (16.69 ± 0.27)	(4.69 ± 0.42) × 10^−4^	(1.47 ± 0.57) × 10^−3^
PM6:PM7:Y6:PC_71_BM	0.859 (0.859 ± 0.001)	26.55 (26.24 ± 0.43)	79.23 (78.7 ± 0.32)	18.07 (17.71 ± 0.23)	(6.53 ± 0.64) × 10^−4^	(1.73 ± 0.16) × 10^−3^
PM7:Y6	0.879 (0.879 ± 0.001)	24.89 (24.79 ± 0.34)	69.10 (67.52 ± 1.25)	15.12 (14.79 ± 0.32)	(3.62 ± 0.56) × 10^−4^	(1.34 ± 0.22) × 10^−3^

The average parameters were calculated from 40 devices, with the area of 0.032 cm^2^. Values outside the parentheses denote the best optimal results.

The stepwise-aligned energy levels lead to interesting device characteristics. The carrier recombination was determined from the dependence of the *J*_SC_ and *V*_OC_ on light intensity, as shown in Supplementary Fig. 5, Supplementary Fig. 8a, and Supplementary Table 3. A slope from *V*_OC_ vs. *P*_light_ of 2 *kT*/*q* should be obtained if monomolecular or trap-assisted recombination dominate^23,24^. The recombination parameter *α*, defined by *J*_SC_ ∝ (*P*_light_)^*α*^, is close to unity, suggesting minimal bimolecular recombination^25^. From the binary to ternary to quaternary blends, *α* increased from ~0.93 to ~0.94 and to ~0.96, and the slope of *V*_OC_ vs. *P*_light_ decreased from 1.34 *kT*/*q* to 1.18 *kT*/*q* and 1.10 *kT*/*q*, respectively, consistent with the change in the FFs. These results indicate that the PM7 donor reduces trap-assisted recombination, due to a better HOMO level alignment, which will be discussed further in the following section. Adding PC_71_BM to the ternary blends leads to a further decrease from the electron transport side. Detailed hole and electron mobilities of the active layers with different thickness were determined using space-charge-limited current with log–log plot (Supplementary Figs. 6 and 7, and Supplementary Table 4). The space-charge-limited region was found with the slope of (2 ± 0.1), which was also fitted according to Mott–Gurney law to obtain mobility values, reflecting electron and hole transport properties^26–28^. It could be seen that higher mobilities correspond to better *J*_SC_ and less recombination, which seems a common phenomenon^29,30^. To further understand the recombination mechanism, transient photovoltage and transient photocurrent were carried out, with details shown in experimental section. Lifetimes under different *V*_OC_ conditions (tuned by changing the light intensity) could be obtained through bi-exponential fitting, as shown in Supplementary Fig. 9a. The lifetime of quaternary device shows a significant enhancement in the whole *V*_OC_ regime, indicating optimized device condition. The charge density as a function of *V*_OC_ for devices is calculated by differential capacitance method, with results shown in Supplementary Fig. 9b, which shows a clear exponential dependence on *V*_OC_, following $n = n_0e^{\gamma V_{\mathrm{OC}}}$, where *n*_0_ is the average charge density in the active layer in dark condition. The value of *γ* (slope of ln(*n*)–*V*_OC_ curve) for each blend was found to give similar results, in which for an ideal semiconductor, a *γ* of *e*/2*kT*, equal to 19.3 V^−1^ at room temperature should be obtained. The deviation can be attributed to the existence of the exponential distribution of the tail states extending to the bandgap of the active layers. The improved charge density in quaternary devices echoes promoted transfer and transport properties, which offset the absorption loss. Derived charge lifetime in the devices as a function of charge density is shown in Supplementary Fig. 9c, following a power law dependence, indicating the nongeminate recombination is the dominating loss channel for carrier density under open-circuit condition. According to the above, nongeminate recombination rate coefficient can be determined, which is defined by $k\left( n \right) = \frac{1}{{\tau \left( n \right)n}}$, as shown in Supplementary Fig. 9d, and the recombination coefficient derived from Langevin theory $k_L = \frac{q}{{\varepsilon _r\varepsilon _0}}( {\mu _n + \mu _p})$ is calculated for comparison^31^. Quaternary devices show the smallest recombination rate coefficient indicating significantly suppressed nongeminate recombination. All of the devices showed a decreased recombination rate coefficient, with two orders of magnitude smaller than the Langevin recombination coefficient. The Langevin recombination rate increases with charge mobility. However, on the basis of detail balanced theory, the charge transport process in photoactive layers is much more complicated at interface, where excitons can dissociate into electrons and holes, meanwhile electrons and holes can meet to generate excitons again or annihilate directly^32^. The much lower recombination coefficient rate compared with Langevin theory indicates a significantly reduced nongeminate recombination loss in devices, contributing to improved devices photoelectric properties. Time-resolved microwave conductivity (TRMC) measurements were performed to characterize the free-charge generation characteristics^33–35^. The ${\varphi\Sigma \mu}$ value at the lowest absorbed flux, where we find excitation intensity-independent recombination dynamics, is used as an indicator of PV potential. As seen in Supplementary Fig. 10a, PM6:Y6 blends peak at a value of 2.3 × 10^−2^ cm^2^ s^−1^ V^−1^. The addition of PM7 and PC_71_BM, slightly increases this value to 2.4 × 10^−2^ cm^2^ s^−1^ V^−1^ for the ternary and 2.9 × 10^−2^ cm^2^ s^−1^ V^−1^ for the quaternary blends, indicating an increased charge generation yield. Shown in Supplementary Fig. 10b are the normalized photoconductivity transient spectra over 500 ns. PM7:Y6 shows the carrier lifetimes increase from 246 to 431 to 460 ns in going from the binary to ternary to quaternary blends. From a local photo-physics perspective, these improved free carrier dynamics, especially in concert with high exciton-to-charge conversion, are consistent with increasingly efficient interfacial exciton dissociation and carrier extraction, which help to improve the long-term stability of the device^36^.

Stability tests were performed for 1000 h under illumination equivalent to 1 sun, and the performance of quaternary devices maintains an 81.0% PCE, with 5.2% *V*_OC_ loss, 5% *J*_SC_ loss, and 10.1% FF loss (Fig. 1g and Supplementary Fig. 11), which is superior than binary devices. Temperature and humidity during the test process are shown in Supplementary Fig. 12 (average 12.5% and 41.5 °C). The storage stability for quaternary devices retain 97.2% PCE after 1000 h aging in dark conditions (Supplementary Fig. 13), underscoring the benefits of the double cascading quaternary blends in enhancing the morphology and operation stability for long-term use. Different cathode interlayers were also investigated during stability test, which yield large variations in performances, suggesting the necessity of cathode interlayer optimization in the future.

### Ultrafast hole transfer and efficient carrier transport along double cascading pathways

**Fig. 2:**
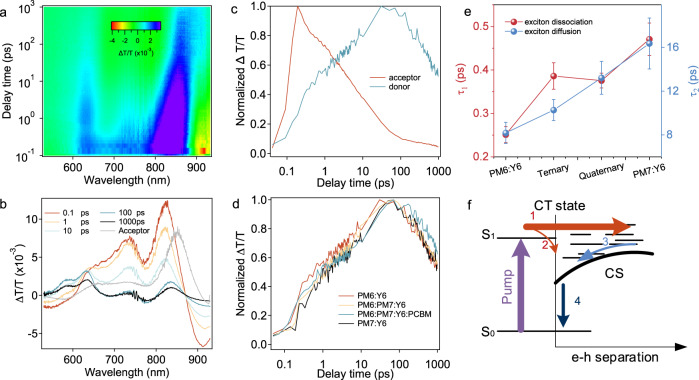
Ultrafast hole transfer and efficient carrier transport along double cascading pathways. **a** Color plot of fs transient absorption spectra of blended film at indicated delay times under 750 nm excitation with a fluence <10 μJ cm^−2^. **b** Representative fs TA spectra of blended films at indicated delay times. **c** TA kinetics in blended films showing the hole transfer process. **d** The hole transfer process in PM6:Y6 (red line), PM6:PM7:Y6 (yellow line), PM6:PM7:Y6:PC_71_BM (blue line), and PM7:Y6 (black line) blended films. **e** Charge-transfer time achieved through multi-exponential fitting for different blended films (the error bar is from the standard error of curve fitting). **f** Schematic of electronic states in an organic solar cell, and the excited state (S_1_) can be directly separated (step 1) or form charge-transfer states (step 2), accompanied with recombination processes (steps 3 and 4).

It is essential to understand the carrier transfer dynamics within the framework of the morphology. Femto-second transient absorption (TA) spectroscopy was used to probe the photoinduced hole transfer dynamics in the multicomponent blends^37–39^. The results are shown in Fig. 2, with the corresponding hole transfer times summarized in Supplementary Table 5. The static absorption peaks for the *D* and *A* are spectrally well separated, so both the spectral and temporal characteristics of hole transfer dynamics can be determined. An excitation wavelength of 750 nm was used to selectively excite Y6. The 2D color plot of TA spectra of PM6:Y6 blend film is shown in Fig. 2a, and a few representative TA spectra at the indicated delay times are shown in Fig. 2b. With the decay of the Y6 bleach peak at 770–860 nm, a few clear bleach peaks at 560–600 nm emerge in the TA spectra, matching well with the absorption features of PM6. The bleach decay process of the photoexcited Y6 agrees with the increase of the PM6 ground state bleach, confirming the ultrafast hole transfer from Y6 to PM6, as shown in Fig. 2c. We can extract the rising kinetic of PM6 bleach to represent the hole transfer process. Herein, the rising kinetic of PM6 bleach is not influenced by Y6 bleach due to well separating of static absorption peaks between PM6 and Y6, providing a clear hole transfer process. We fit the donor kinetics with a bi-exponential function. The hole transfer process in the four blends (PM6:Y6, PM6:PM7:Y6, PM6:PM7:Y6:PC_71_BM, and PM7:Y6) show a fast component with *τ*_1_ of ~0.25, ~0.39, ~0.37, and ~0.47 ps, and a slow component *τ*_2_ of 8.18, 10.28, 13.22, and 16.36 ps, respectively (Fig. 2d, e), and their relative contributions are shown in Supplementary Table 5. The former fast component *τ*_1_ can be assigned to the ultrafast exciton dissociation of Y6 at the donor–acceptor interface and the latter to the diffusion of excitons in Y6 toward interface before dissociation^40–42^. The interfacial exciton separation is more than one order of magnitude faster in comparison to the exciton diffusion. The trend in the hole transfer lifetime and the hole transfer efficiency is consistent with the values of HOMO offsets or driving force (Supplementary Fig. 14), which points out the direction of device operating principle. The presence of PM7 in the BHJ blends reduces the driving force, aligning the cascading energy levels to ensure better transport and an increase in the *J*_SC_, a trade-off with the hole transfer rate. PC_71_BM does not perturb the hole transfer process, suggesting close interactions between Y6 and PM6/PM7. Such results also agree well with the observation that no PM6:PC_71_BM CT emission is seen in quaternary blends. While most studies focus on increasing absorption with the addition of more components, our findings indicate that a detailed balance between the driving force and hole transfer rate is equally important to refine the carrier generation and extraction to generate high *J*_SC_ in OSCs. We compared the polaron decay dynamics of the quaternary blend films at different pump fluences to probe the charge recombination mechanism (Supplementary Fig. 15). The difference in fluence dependence of recombination dynamics suggests that carriers recombine via the nongeminate recombination with small amount of geminate recombination (1%), indicating of small amount of CT states formation. Time-resolved photoluminescence (TRPL) measurements were further performed. To avoid the influence of Y6 fluorescence quenching, we only probed 550–650 nm and pump at 515 nm in TRPL measurement. As illustrated in Supplementary Fig. 16 and Supplementary Table 6, PM6:PM7 blended film presents much smaller fluorescence lifetime (*τ* = 672.4 ± 9.3 ps) compared with PM6 (1483.6 ± 11.7 ps) and PM7 (1521.7 ± 13.1 ps) neat films, indicating electron transfer channel between PM6 and PM7. After blending Y6, the fluorescence lifetime is significantly decreased with a time constant of 73.9 ± 1.23 ps, indicating a highly efficient electron transfer in the PM6:PM7:Y6 heterojunction. From the above results, a picture of the mechanism emerges, as shown in Fig. 2f. The photon excitation first drives ultrafast and large amounts of free carriers accompanied with nongeminate recombination, biasing out the weak interfacially bound CT states (which are quite close in energies), along with a density-dependent recombination process (step 4).

### Energy loss optimization induced by CT energy management

**Fig. 3:**
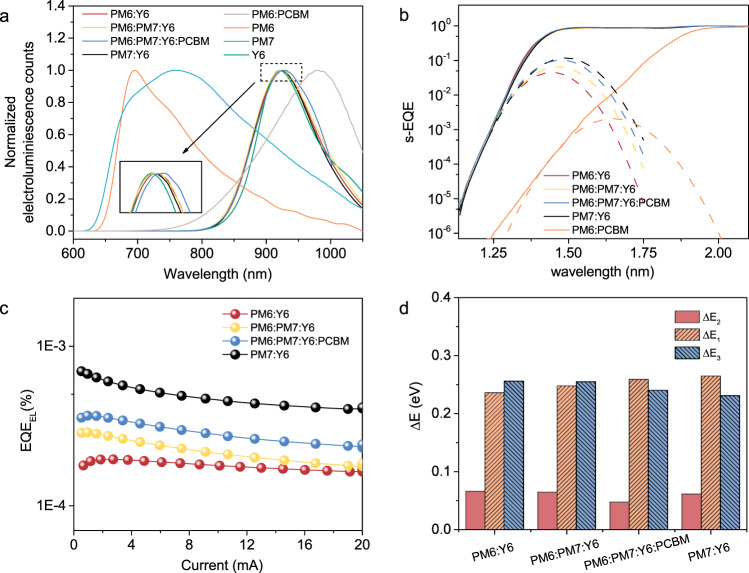
Energy loss analysis. **a** Electroluminescence spectra of devices based on the pristine and blended films. **b** s-EQE and **c** EQE-EL of the blended devices. **d** Energy loss histogram, including ∆*E*_1_, ∆*E*_2_, and ∆*E*_3_ of blended devices.

In solar cell devices, the *V*_OC_ decreases in going from quaternary to ternary to binary blends. This results from the energy level management, driving force, and energy loss (*E*_loss_). Highly sensitive EQE (s-EQE), electroluminescence (EL), and electroluminescence quantum efficiency (EQE-EL) measurements were performed to investigate energy loss channels^43–45^. Two energy loss sectors, charge generation (∆*E*_2_ = *E*_g_–*E*_CT_) and charge recombination losses (*E*_CT_–*qV*_OC_) were considered. The charge recombination loss could be further traced to radiative (∆*E*_1_) and non-radiative (∆*E*_3_) recombinations^46^. The *E*_CT_ could be obtained by fitting the sub-gap absorption of the corresponding s-EQE curve (Fig. 3b and Supplementary Fig. 17) following the Marcus theory^43,47^. The CT energy of the binary PM6:Y6 blend was 1.34 eV, yielding a ∆*E*_2_ = 0.066 eV. PM7:Y6 blend showed a CT energy of 1.37 eV, with ∆*E*_2_ = 0.061 eV. Ternary and quaternary blends showed the CT energies of 1.35 and 1.36 eV, respectively. The lowest ∆*E*_2_ of 0.048 eV was obtained for quaternary films, resulting in a higher *V*_OC_. The deeper HOMO of PM7 leads to a reduced hole transfer driving force but smaller energy loss, providing one avenue for device optimization, with an optimized ternary blend composition of 20% PM7. The PM6:PC_71_BM mixture has a CT energy of 1.49 eV, higher than the bandgap of Y6, and thus would not introduce a low level CT state to trap electrons. Consequently, adding PC_71_BM to the ternary blends redefines the acceptor LUMO at a higher level, which further improves *V*_OC_.

Figure 3a shows the normalized EL spectra of devices based on the pure materials and the BHJ films. PM6:Y6 blends show a single EL emission peak at 924 nm, similar to that of a Y6 film (920 nm). When the applied current was increased from 1 to 5 mA, no EL change was observed (Supplementary Fig. 19). Thus, the CT states, if present, have a very low density. PM6:PC_71_BM blends have a CT emission at 980 nm, well below the emission in the binary mixtures. However, in the quaternary blends, the EL is dominated by Y6, eliminating the PM6: PC_71_BM CT states. Therefore, the BHJ blends can be viewed as simple OLED devices, with PM6/PM7 and PC_71_BM acting as hole and electron transporting layers, and emission happens at the Y6 acceptor or PM6:Y6 interfaces. Y6, then, functions as both a photovoltaic and EL material. The energy losses due to radiative recombination (∆*E*_1_) of charge carriers can be calculated using the fit parameters from the s-EQE spectra (Fig. 3b and Supplementary Fig. 17), and the losses due to the non-radiative recombination (∆*E*_3_) were quantified by measuring the EQE-EL^48^. As shown in Fig. 3c, the emission efficiency of PM7:Y6 blend was 1.33 × 10^−2^%, much higher than that of PM6:Y6 blend (5.05 × 10^−3^%). Thus, for ternary blends, the additional 20% PM7 actually reduced the energy loss caused by non-radiative recombination. The quaternary blends show a higher emission efficiency of 9.37 × 10^−3^% in comparison to the ternary film, representing a decrease in ∆*E*_3_ to 0.240 eV. Non-radiative recombination can be calculated from ∆*E*_3_ = −*kT*·ln(EQE_EL_). Consequently, it is important to maximize EQE_EL_ to minimize ∆*E*_3_, and in the current case, the double cascading energy level alignment plays an important role. Different contributions to energy loss are shown in Fig. 3d and summarized in Supplementary Table 7. The quaternary blends showed the lowest total energy loss of 0.548 eV, in comparison to the other blends (0.567 eV for PM6:Y6, 0.553 eV for PM7:Y6, and 0.568 eV for ternary blends). We attribute the elevated *V*_OC_ of the quaternary devices to the smallest *E*_g_–*E*_CT_ energy offset and the suppressed non-radiative recombination losses, due to the addition of PM7 and PC_71_BM.

### Thin film morphology of double cascading blends

**Fig. 4:**
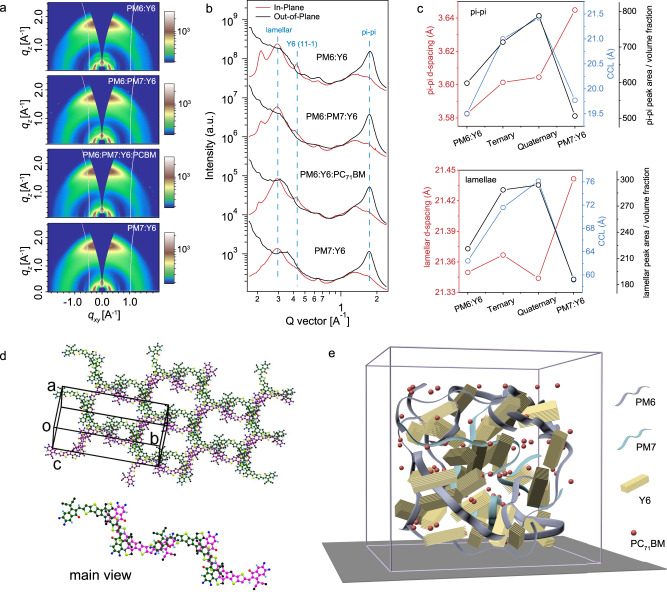
Morphology optimization of thin films. **a** 2D GIXD patterns of the binary, ternary and quaternary blends. **b** Out-of-plane (black) and in-plane (red) linecut profiles of the 2D GIXD data. **c** *D*-spacing (red symbol), CCL (blue symbol) and peak area/volume fraction (black symbol) of pi–pi and lamellae diffraction peak for blended films with different composition. **d** Molecular packing sketch map of Y6 crystal. **e** The arrangement sketch of molecules in quaternary blended films.

The structure of the neat and BHJ thin films were determined using grazing incidence wide-angle X-ray diffraction (GIWAXS), the results of which are shown in Fig. 4 and Supplementary Fig. 20. The PM6 donor assumed a dominant face-on orientation, with a broad (100) reflection in the in-plane (IP) direction at 0.28 Å^−1^ and a π–π stacking peak in the out-of-plane (OOP) direction at 1.69 Å^−1^. The crystal coherence lengths (CCLs) for (100) and (010) were 5.27 and 1.56 nm, respectively, as determined using the Scherrer analysis^49,50^. PM7 had a similar diffraction profile, with a (100) reflection in the IP direction at 0.29 Å^−1^ and (010) reflection in the OOP direction at 1.66 Å^−1^ with CCLs of 4.52 and 1.31 nm, respectively. Y6 films showed a π–π stacking peak at 1.75 Å^−1^ in the OOP direction and a lamellar stacking peaks at 0.27 Å^−1^ in the IP direction. However, the banana-shaped Y6 molecules pack in a unique manner so that they can overlap by end group π–π stacking to form a polymer-like conjugated backbone, and the lamellae packing is assigned to the (110) lattice plane, which is shown in Fig. 4d and Supplementary Fig. 21. Thus, Y6 assumes a tilted molecular orientation where the polymer-like backbone is tilted normal to surface, which is more efficient for charge transport.

A summary of the 2D and linecut GIWAXS profiles are shown in Fig. 4a,b, respectively. For the PM6:Y6 blends, Y6 showed well-defined IP (020) and (11-1) lattice reflections at 0.21 and 0.42 Å^−1^. PM7:Y6 blends show weak crystalline order, with the Y6 (11-1) and (020) diffraction peaks absent. Even though PM6 and PM7 have similar chemical structures, PM7 interacts differently with Y6 and retards Y6 crystallization, such that only the Y6 (110) stacking can be seen. In ternary blends, the intensities of the reflections at 0.23 and 0.42 Å^−1^ decreased significantly. In the quaternary blends, the Y6 reflections at 0.23 and 0.42 Å^−1^ were quite weak, indicating that PC_71_BM also disrupts the packing of Y6. The polymer lamellar and Y6 (110) reflections could not be separated, and they were used in sum to estimate the lamellar ordering of the BHJ thin film. The polymer and Y6 π–π reflections are summarized in Supplementary Table 8. Figure 4c shows parameters derived from the lamellar and π–π stacking peaks of the different BHJ thin films. The quaternary blend showed the largest CCL and peak area for both the lamellar and π–π stacking peaks, indicating that the overall crystallinity and crystal quality are improved for the quaternary blends, which improves carrier transport pathways. The Y6 (020) peak showed a decrease in the peak area, but an increase in the CCL in going from the binary to ternary to quaternary blends, as shown in Supplementary Fig. 22 and Supplementary Table 9. Consequently, the crystallization behavior of Y6 changed in the blends. The loss of primary axis coherence and intensity in Y6, but improvement in the (110) and π–π stacking reflect an extended polymer-like conjugated backbone by adopting a twisted or screw-like packing in the multicomponent blends, providing a pathway for electron transport. A schematic of the molecular packing in the blend films is illustrated in Fig. 4e. The intimate mixing of PM6 and PM7 (as indicated by the linear dependence of the *V*_OC_ on concentration) results in the formation of a homogeneous polymer-rich phase embedded in a fibrillar network. The difference in the interactions of PM6 and PM7 with Y6 optimizing the crystallization of Y6. PC_71_BM addition does not perturb the morphological framework of PM6:Y6 and is distributed uniformly, as evidenced by the absence of any feature characteristic of PC_71_BM aggregation. Thus, the plasticizing nature of PC_71_BM aids in the overall ordering, improving both electron and hole mobility, and a higher FF.

BHJ thin film phase separation was visualized using transmission electron microscopy (TEM). As shown in Supplementary Fig. 23, all the BHJ thin films showed evidence of phase separation on the tens of nanometers length scale. Resonant soft x-ray scattering (Supplementary Fig. 24) for PM6:Y6 and PM6:PM7:Y6 blends yielded an interference at a length scale of ~60 nm. The quaternary blends with different PC_71_BM loadings (Supplementary Fig. 24b) show a shoulder gradually developing into a well-defined interference at higher PC_71_BM loadings, suggesting that uniform distribution of PC_71_BM in the mixed region enhances the scattering contrast. The uniform distribution of PC_71_BM in the mixture indicates that the close interactions with the other amorphous components produces a unique electronic structure with improved electron transport channels, where excited electrons in the donors can transfer onto the “LUMO” of mixed domain that are rapidly extracted. Though the HOMO of PC_71_BM is much deeper than that of Y6, a homogeneous mixing provides good contacts with the donor materials making exciton harvesting by PC_71_BM and Y6 efficient.

## Discussion

**Fig. 5:**
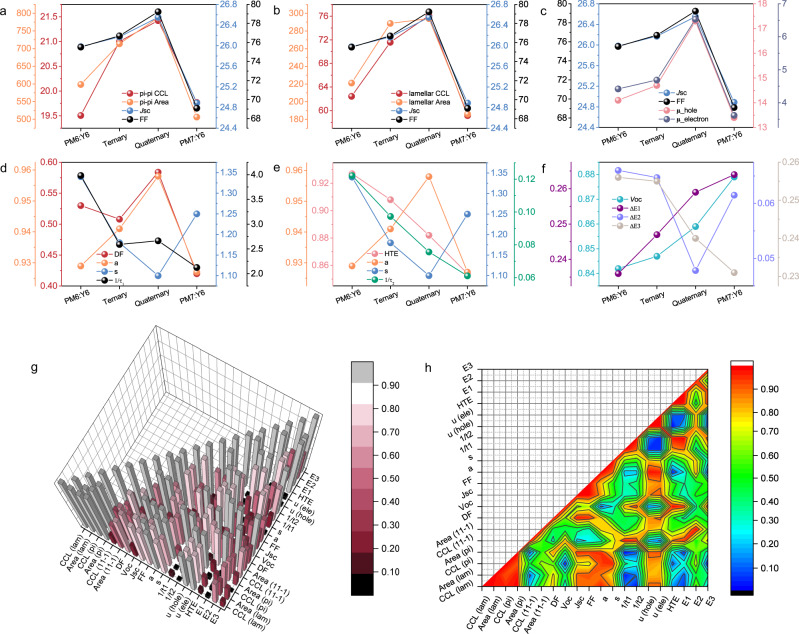
Correlation of structure, photon-to-electron process, and device performance. **a**–**f** Multidimensional correlation analysis of structure–property relationships (the parameter units are following previous data). **g**, **h** Person correlation analysis of major device and structure parameters (color bar and height both represent person correlation coefficients, CCL is the abbreviation of crystal coherence length, DF is the abbreviation of driving force, HTE is the abbreviation of hole transfer efficiency, E1–E3 represent energy loss).

The results show the importance of achieving a detailed balance between morphology, energy loss reduction, and ultrafast charge transfer kinetics in double cascading quaternary blends. The double donor strategy yields a terraced HOMO energy level alignment that facilitates hole transport in the BHJ blends. Adding PC_71_BM that is uniformly mixed with Y6 produces a terraced LUMO energy level alignment, and in the HOMO levels, the longer exciton diffusion times, in comparison to the splitting times, lead to a high probability that a suitable donor/acceptor interface can be found to split excitons. The close contact between Y6 and PM7 provides an important channel where Y6 trapped exciton or hole carriers can be extracted. Such a unique double cascading energy level simultaneously improves the *V*_OC_, *J*_SC_, and FF. A more thorough correlation between the structure, photon–electron processes, and device performances needs to be quantified to establish a solid structure–property relationship. Shown in Fig. 5a, b are the correlations between the structural and device characteristics, Fig. 5c–e shows relationships between charge transfer, transport, and performance, and Fig. 5f, the correlation between energy loss and *V*_OC_. A quantitative analysis was performed using the Pearson correlation method^51,52^, as shown in Fig. 5g, h in 3D and 2D. A correlation factor of 1, indicates a direct correlation, with decreasing values reflecting a loss in correlation. The Pearson correlation coefficient matrix is shown in Supplementary Table 10. It is seen that crystallization is the major factor that accounts for *J*_SC_ and FF in photovoltaic devices, thus the quaternary blends reach the maximum value. Mobilities (*µ*_h_ and *µ*_e_) are highly dependent on thin film crystallinity. 1/*τ*_1_ shows a strong correlation with the driving force, indicating its strong influence on exciton separation, while the trend in driving force matches well with recombination parameters, suggesting an important mechanism correlating efficient charge transfer and extraction. *V*_OC_ should show an inverted correlation with energy loss, and ∆*E*_3_, originating with non-radiative recombination, shows the most significant influence. Therefore, a detailed balance between morphology optimization, charge transfer efficiency, and energy loss channels controls the device output. These observations suggest a new strategy using double cascading quaternary blends for device optimization. The material properties and device performance parameters are highly interconnected, and optimizing only one parameter is not sufficient to optimize performance. Binary mixtures are effective for material screening, and ternary mixtures bring complementary absorption and energy level management into the device design, but quaternary mixtures enable a fine-tuning of both the morphology and electronic structure simultaneously. Fine-tuning such quaternary mixtures introduces a new strategy to achieve higher efficiency OPVs. We extended the double cascading quaternary mixture strategy to other well-studied NFA OPV systems (Supplementary Fig. 25 and Supplementary Table 11), with similar results been obtained as seen in PM6:PM7:Y6:PC_71_BM mixture, underscoring the generality of this approach.

Here, we designed multicomponents blends of polymer donors PM6 and PM7, a non-fullerene small-molecule acceptor Y6, and a fullerene acceptor PC_71_BM. An average PCE of 16.69% was obtained when 80% PM6 and 20% PM7 were blended with Y6. The addition of the fourth component PC_71_BM significantly increased the average efficiency output to 17.71% (PCE_max_ of 18.07%), with a slightly improved *J*_SC_ of 26.55 mA cm^−2^, which could be ascribed to a fine balance between light absorption and charge extraction. The elevated *V*_OC_ (0.859 V) of the quaternary device was attributed to the smallest *E*_g_–*E*_CT_ energy offset and the suppressed non-radiative recombination losses after the addition of PM7 and, especially, PC_71_BM. An FF of over 0.79 was obtained, since an optimized morphology formed with balanced crystallization features that ensured better charge transport, as shown in Supplementary Fig. 26 that summarizes the holistic strategy in OPV efficiency optimization.

## Methods

### Device fabrication

Organic solar cell devices with ITO/PEDOT:PSS/active layer/PFNDI-Br/Ag regular structures were fabricated according to the following procedure. Patterned ITO glass substrates were sequential cleaned by ultrasonicating in acetone, detergent, deionized water, and isopropyl alcohol for 15 min each and then dried under 80 °C. The precleaned substrates were treated in an ultraviolet–ozone chamber for 15 min, then a ~40 nm thick PEDOT:PSS (Clevious P VP AI 4083 H. C. Stark, Germany) thin film was deposited onto the ITO surface by spin-coating and baked at 150 °C for 20 min. The blend solution with different mixing ratio (14 mg mL^−1^ in total) in CF (with 0.5% CN solvent additive) was stirred at 25 °C for 120 min in advance, and then spin-coated on top of the PEDOT:PSS layer. The prepared films were treated with thermal annealing at 85 °C for 6 min. After cooling to room temperature, a ~5 nm thick of PFNDI-Br (0.5 mg mL^−1^) was spin-coated on the top of active layer. Then, those samples were brought into to an evaporate chamber and a 140 nm thick silver layer was thermally evaporated on the PFNDI-Br layer at a base pressure of 1 × 10^−6^ mbar. The evaporation thickness was controlled by SQC-310C deposition controller (INFICON, Germany). Ten devices were fabricated on one substrate and the active area of each device was 0.032 cm^2^ defined by a shadow mask.

### Reporting summary

Further information on research design is available in the Nature Research Reporting Summary linked to this article.

## Supplementary information

Supplementary Information

Reporting Summary

Solar Cells Reporting Summary

## Data Availability

The data that support the plots within this paper and other finding of this study are available from the corresponding authors upon reasonable request.
