# Supplementary material for: Single-layered organic photovoltaics with double cascading charge transport pathways: 18% efficiencies
Source: Nat Commun. 2021 Jan 12;12:309. doi: 10.1038/s41467-020-20580-8 (PMC7803987; doi:10.1038/s41467-020-20580-8)
Supplement: Supplementary file 1 — Supplementary Information [file 41467_2020_20580_MOESM1_ESM.pdf]

Single-layered organic photovoltaics with double cascading charge transport pathways: 18 % efficiencies

Ming Zhang[1], Lei Zhu[1], Guanqing Zhou[1], Tianyu Hao[1], Chaoqun Qiu[1], Zhe Zhao[1], Qin Hu[2], Bryon W. Larson[3], Haiming Zhu[4], Zaifei Ma[5], Zheng Tang[5], Wei Feng[6], Yongming Zhang[1,6], Thomas P. Russell[2] and Feng Liu[1,6]*

[1]Frontiers Science Center for Transformative Molecules, In-situ Center for Physical Science, and Center of Hydrogen Science, School of Chemistry and Chemical Engineering, Shanghai Jiao Tong University, Shanghai 200240, P. R. China.

[2]Department of Polymer Science and Engineering, University of Massachusetts, Amherst, MA 01003, USA

[3]Chemistry & Nanoscience Department, National Renewable Energy Laboratory, Golden, Colorado 80401, USA

[4]Department of Chemistry, Zhejiang University, Hangzhou 310027, P. R. China.

[5]Center for Advanced Low-dimension Materials, State Key Laboratory for Modification of Chemical Fibers and Polymer Materials, College of Materials Science and Engineering, Donghua University, Shanghai 201620, P. R. China.

[6]State Key Laboratory of Fluorinated Functional Membrane Materials and Dongyue Future Hydrogen Energy Materials Company, Zibo City, Shandong Province 256401, P. R. China.

These authors contributed equally: Ming Zhang, Lei Zhu, Guanqing Zhou

*email: fengliu82@sjtu.edu.cn

Supplementary Methods

Materials. All reagents and chemicals were purchased from commercial sources (Aldrich or Acros) without further purification. PM6 and Y6 were purchased from Solarmer Company. PM7 were purchased from Shanghai Vizuchemical Scientific Company. $PC_{71}BM$ was purchased from American Dye Source, Inc. The silicon nitride membrane is from Clean -SiN Company.

General methods. UV-vis absorption spectra were recorded on a Shimadzu spectrometer model UV-1800 with films on the quartz plates at room temperature. The morphologies of the BHJ thin films were characterized by transmission electron microscopy (TEM, JEM-ARM200F, Japan). The GIWAXS characterization of the thin films was performed at the Advanced Light Source (Lawrence Berkeley National Laboratory) on beamline 7.3.3, with the incidence angle was 0.16°, and the beam energy of 10 keV. Samples were prepared under optimal conditions on the Si/PEDOT:PSS substrates. RSoXS was performed at beamline 11.0.1.2 (Lawrence Berkeley National Laboratory) with a bean energy of 284.2eV. Samples were prepared under optimal conditions on the Si/PEDOT:PSS substrates, then placed in water and transferred to a silicon nitride window.

Device Characterization. The current-voltage (*J-V*) curves were measured with Keithley 2400 Source under the illumination of AM 1.5G irradiation (100 mW cm^{-2}) using a 150 W solar simulator (DM-40S3, SAN-EI ELECTRIC, Japan) in glove box at room temperature. The light intensity was determined by a 2 × 2cm^2 standardized monosilicon cell (Oriel PN 91150V, Newport, USA.) calibrated by the National Renewable Energy Laboratory (NREL). The external quantum efficiency (EQE) measurement was performed using an Enlitech EQE

system (Enlitech QE-M110) with a Si diode as reference cell. The $J–V$ curves were measured along the forward scan direction from -0.2 to 1.0 V or the reverse scan direction from 1.0 to -0.2 V, yielding identical results. The scan speed and dwell times were fixed at 0.015 V s^{-1} and 20 ms, respectively. White LEDs, and an ultraviolet LED (centred at 365 nm) were used as the light source for stability test, the intensity of which was controlled to consistently provide an output of 100 mW/cm^2. The LED intensity that produced a photocurrent equivalent to J_{SC} under AM1.5G illumination (J_{SC}, AM1.5G) was defined as equivalent to 1-Sun intensity.

SCLC mobility measurements. The electron-only devices were fabricated with ITO/ZnO/Active Layer/ZnO/Ag structures and hole-only devices were fabricated with ITO/PEDOT:PSS/Active Layer/MoO_x/Al structures. The space charge limited current (SCLC) mobility was calculated according to the Mott-Gurney square law $J = 9\varepsilon_r\varepsilon_0\mu V^2/8L^3$, where J is the current density, ε_r is the relative dielectric constant of the transport medium component, ε_0 is the vacuum permittivity, μ is the electron or hole mobility, V is the effective voltage, and L is the thickness of active layer.

Ultraviolet photoelectron spectroscopy measurement. Ultraviolet Photoelectron Spectroscopy analysis was conducted using an AXIS Ultra DLD spectrometer (Kratos Analytical Inc., Mancheser, UK) with a He discharge UV lamp with He I radiation (incident photo energy, 21.22 eV), a hemispherical analyzer operating in the fixed analyzer transmission mode and the standard aperture (analysis area: 0.3 mm * 0.7 mm).

Transient photovoltage (TPV) and photocurrent (TPC) measurement. The lifetime of carriers can be measured by the transient photovoltage measurement. The background

illumination was provided by a normal LED light source, and pulsed light was provided by arbitrary wave generator (AFG322C, Tektronix). The photovoltage traces were registered by the oscilloscope (AFG322C, Tektronix). The photocurrente traces were registered with the resistance of 50 Ω, switching open-circuit mode to short-circuit mode. The integrated TPC signal provides a measure of the total charge generated by the laser pulse (ΔQ). Empirically, the differential capacitance values are found to follow the exponential dependence on the open-circuit voltage given by $C = \frac{\Delta Q}{\Delta V} = C_0 exp(\gamma V_{OC}) + D$, and so the charge-carrier density as a function of V_{OC} is given by treating the device as a parallel-plate capacitor and integrating with respect to voltage, as $n = \frac{1}{Aed}\int_{-\infty}^{V_{OC}} C_0 \exp(\gamma V) dV$, where A is the active layer area, and d is the active layer thickness. Then, the recombination rate coefficient can be determined, which is defined by $k(n) = \frac{1}{\tau(n)n}$.

Electroluminescence measurement. Electroluminescence spectrum measurement was conducted by direct-current meter (PWS2326, Tectronix) to provide bias voltage for the test device, and the electroluminescence emissions were recorded by the fluorescence spectrometer (KYMERA-328I-B2, Andor technology LTD).

EQE$_{EL}$ measurement. The EQE$_{EL}$ was recorded with an in-house-built system comprising a standard silicon photodiode 1010B, Keithley 2400 source meter (for supplying voltages and recording injected currents), and Keithley 6482 picoammeter (for measuring the emitted light intensity).

Highly sensitive EQE (s-EQE). The halogen light source (LSH-75, Newport) passed through the monochromator(CS260-RG-3-MC-A, Newport) to form monochromatic light, which was focused on the device to generate electrical signals. Signals were finally collected by the

front-end current amplifier (SR570, Stanford) and phase-locked amplifier (Newport). A corrected silicon solar cell (S1337-1010BR) was used as a standard detector.

Transient absorption spectroscopy (TA). For femtosecond transient absorption spectroscopy, the fundamental output from Yb:KGW laser (1030 nm, 220 fs Gaussian fit, 100 kHz, Light Conversion Ltd) was separated to two light beam. One was introduced to NOPA (ORPHEUS-N, Light Conversion Ltd) to produce a certain wavelength for pump beam (here we use 550 and 750 nm, 30 fs pulse duration), the other was focused onto a YAG plate to generate white light continuum as probe beam. The pump and probe overlapped on the sample at a small angle less than 10°. The transmitted probe light from sample was collected by a linear CCD array.

Time-resolved microwave conductivity (TRMC). TRMC measurements were performed on thin films of only the active layer (no electrodes or transport layers), using a pulsed Q-switched neodymium yttrium-aluminium-garnet (Nd:YAG) laser (3-5 ns pulses, 10 Hz repetition rate, Continuum Powerlite) and an optical parametric oscillator (Continuum Panther) pumped by the third harmonic (355 nm) of the Nd:YAG laser. Neutral density filters enabled the adjustment of the pump fluence by up to four orders of magnitude. A calibrated Schottky barrier diode (~1 ns response time) measured time-dependent changes in the microwave probe power, and an oscilloscope with subnanosecond resolution tracked the transient behaviour of changes in the microwave power. Although these types of measurements often employ a partially transparent (to microwaves) metal iris to create a resonant microwave cavity that enhances signal strength at the expense of time resolution, the signal here was sufficiently strong that we chose to omit the iris and maintain the full temporal resolution afforded by the

~4 ns optical pump.

Supplementary Tables

Supplementary Table 1. Photovoltaic parameters with different PM6:PM7 ratios for ternary devices under illumination of AM 1.5G, 100 mW cm^{-2}.[a]

PM6:PM7:Y6	V_{OC} (V)	J_{SC} (mA·cm^{-2})	FF (%)	PCE[b] (%)
1:0:1.2	0.842<br>(0.842±0.001)	25.98<br>(25.67±0.19)	75.52<br>(74.91±0.66)	16.52<br>(16.30±0.11)
0.8:0.2:1.2	0.848<br>(0.847±0.001)	26.17<br>(25.72±0.31)	76.70<br>(75.91±0.72)	17.02<br>(16.69±0.27)
0.6:0.4:1.2	0.854<br>(0.852±0.002)	25.86<br>(25.77±0.12)	74.68<br>(73.86±0.75)	16.49<br>(16.18±0.35)
0.4:0.6:1.2	0.861<br>(0.861±0.001)	25.60<br>(25.01±0.57)	73.09<br>(71.22±1.63)	16.11<br>(15.97±0.11)
0.2:0.8:1.2	0.875<br>(0.875±0.001)	25.29<br>(24.93±0.19)	70.86<br>(69.52±0.84)	15.68<br>(15.33±0.29)
0:1:1.2	0.879<br>(0.879±0.001)	24.89<br>(24.79±0.34)	69.10<br>(67.52±1.25)	15.12<br>(14.79±0.32)

[a]The device architecture is ITO/PEDOT:PSS/PM6:PM7:Y6/PFNDI-Br/Ag; D:A=1:1.2, D concentration = 6.5 mg ml^{-1} in CF with 0.5% 1-CN, following with 85°C TA treatment. [b]The average values are obtained from over 40 devices.

Supplementary Table 2. Photovoltaic parameters with different $PC_{71}BM$ ratios for quaternary devices under illumination of AM 1.5G, 100 mW cm^{-2}.[a]

PM6:PM7:Y6:$PC_{71}BM$	V_{OC} (V)	J_{SC} (mA·cm^{-2})	FF (%)	PCE [b] (%)
0.8:0.2:1.2:0	0.848<br>(0.847±0.001)	26.17<br>(25.72±0.31)	76.70<br>(75.91±0.72)	17.02<br>(16.69±0.27)
0.8:0.2:1.2:0.25	0.859<br>(0.859±0.001)	26.55<br>(26.24±0.43)	79.23<br>(78.7±0.32)	18.07<br>(17.71±0.23)
0.8:0.2:1.2:0.5	0.859<br>(0.859±0.001)	26.16<br>(25.60±0.69)	75.92<br>(75.92±0.93)	17.06<br>(16.79±0.38)
0.8:0.2:1.2:0.75	0.878<br>(0.878±0.001)	25.83<br>(25.13±0.52)	71.91<br>(71.44±0.56)	16.31<br>(16.16±0.27)

[a]The device architecture is ITO/PEDOT:PSS/PM6:PM7:Y6:$PC_{71}BM$/PFNDI-Br/Ag; D concentration = 6.5 mg ml^{-1} in CF with 0.5% 1-CN, following with 85°C TA treatment. [b]The average values are obtained from over 40 devices.

Supplementary Table 3. Parameters of light intensity experiment through linear fitting.

**Blend**	α	**s**
PM6:Y6	0.929±0.006	(1.34±0.07) kT/q
PM6:PM7:Y6	0.941±0.011	(1.18±0.05) kT/q
PM6:PM7:Y6:$PC_{71}BM$	0.958±0.004	(1.10±0.04) kT/q
PM7:Y6	0.927±0.004	(1.25±0.10) kT/q

α and s represent the parameters fitted from J_{SC} versus light intensity and V_{OC} versus light intensity, respectively.

Supplementary Table 4. Mobilities obtained from SCLC measurements.

Blend	Thickness(nm)	$\mu_{electron}$ ($cm^{-1}s^{-1}V^{-1}$)	μ_{hole}($cm^{-1}s^{-1}V^{-1}$)
PM6:Y6	110	(2.33±0.29) $\times 10^4$	(1.03±0.14) $\times 10^3$
	140	(4.43±0.34) $\times 10^4$	(1.38±0.37) $\times 10^3$
	180	(7.38±0.45) $\times 10^4$	(2.42±0.21) $\times 10^3$
PM6:PM7:Y6	110	(4.31±0.18) $\times 10^4$	(9.70±0.42) $\times 10^4$
	140	(4.70±0.35) $\times 10^4$	(1.51±0.29) $\times 10^3$
	180	(7.41±0.53) $\times 10^4$	(2.17±0.19) $\times 10^3$
PM6:PM7:Y6:PCBM	110	(3.60±0.17) $\times 10^4$	(1.44±0.23) $\times 10^3$
	140	(6.48±0.32) $\times 10^4$	(1.86±0.34) $\times 10^3$
	180	(8.99±0.54) $\times 10^4$	(2.29±0.24) $\times 10^3$
PM7:Y6	110	(2.40±0.24) $\times 10^4$	(7.06±0.34) $\times 10^4$
	140	(3.82±0.41) $\times 10^4$	(1.19±0.48) $\times 10^3$
	180	(5.07±0.64) $\times 10^4$	(1.92±0.14) $\times 10^3$

Supplementary Table 5. Lifetime of the hole transfer process in blended films. The data was achieved through biexponential fitting.

**Blend**	A_1	**τ_1 (ps)**	A_2	**τ_2 (ps)**
PM6:Y6	0.51±0.0191	0.25104±0.0183	0.42±0.0183	8.183±0.968
PM6:PM7:Y6	0.45±0.0165	0.38602±0.0305	0.49±0.0179	10.278±0.970
PM6:PM7:Y6:$PC_{71}BM$	0.42±0.0153	0.37552±0.0170	0.48±0.0196	13.219±1.501
PM7:Y6	0.46±0.0176	0.47073±0.0374	0.49±0.0187	16.361±2.331

Supplementary Table 6. Lifetime derived from TRPL measurements.

**Blend**	**τ (ps)**
PM6	1483.6±11
PM7	1521.7±13
PM6:PM7	672.4±9.3
PM6:Y6	73.3±1.58
PM6:PM7:Y6	73.9±1.23
PM6:PM7:Y6:$PC_{71}BM$	76.4±1.77
PM7:Y6	72.6±1.72

Supplementary Table 7. Energy loss details for different blends.

Conditions	V_{OC} (V)	E_{gap} (eV)	ΔE (eV)	ΔE_1 (eV)	ΔE_2 (eV)	ΔE_3 (eV)	E_{CT} (eV)	λ (eV)
PM6:Y6	0.842	1.406	0.564	0.236	0.0660	0.256	1.340	0.109
PM6:PM7:Y6	0.847	1.415	0.567	0.247	0.0647	0.255	1.349	0.123
PM6:PM7:Y6:$PC_{71}BM$	0.859	1.408	0.548	0.259	0.0478	0.240	1.361	0.122
PM7:Y6	0.879	1.420	0.541	0.264	0.0615	0.231	1.373	0.126

Supplementary Table 8. Structure parameters for different blended films. All four blends show face-on orientation, and polymer lamellae and Y6 (110) peaks mainly locate in IP direction, while pi-pi stacking peak in OOP direction. Position, FWHM and peak area are available through multi-peak fitting and d-spacing, CCL can be calculated by Scherrer Equation.

Conditions	Lamellae D-Spacing (Å)	Lamellae CCL (Å)	Lamellae Peak Area	pi-pi D-Spacing(Å)	pi-pi CCL (Å)	pi-pi Peak Area
PM6:Y6	21.35	62.43	221.16 ± 3.15	3.582	19.50	598.59 ± 12.52
PM6:PM7:Y6	21.37	71.58	288.56 ± 5.42	3.601	21.00	713.84 ± 7.34
PM6:PM7:Y6:$PC_{71}BM$	21.34	76.12	294.06 ± 5.12	3.604	21.42	787.64 ± 4.98
PM7:Y6	21.44	59.15	186.07 ± 12.73	3.645	19.77	506.41 ± 13.26

Note: The error of d-spacing and CCL both less than 0.01 Å, which is due to the statistical properties of the scattering technology itself.

Supplementary Table 9. Structure parameters from for Y6 (11-1) and (020) peaks.

Conditions	(11-1) D-Spacing (Å)	(11-1) CCL (Å)	(11-1) Peak Area	(020) D-Spacing (Å)	(020) CCL (Å)	(020) Peak Area
PM6:Y6	14.73	127.28	21.52 ± 0.52	29.24	282.79	29.58 ± 0.43
PM6:PM7:Y6	15.03	68.14	25.38 ± 0.74	29.31	324.01	12.57 ± 0.13
PM6:PM7:Y6:$PC_{71}BM$	14.95	55.40	58.54 ± 1.33	30.53	517.73	2.86 ± 0.01
PM7:Y6[a]	15.23	42.65	34.94 ± 0.34			

[a]For PM7:Y6 blends, Y6 (020) peak totally disappeared, and corresponding parameters was not available.

Supplementary Table 10. Person correlation coefficients matrix.

	CCL_{lam}	$Area_{lam}$	CCL_{pi}	$Area_{pi}$	CCL_{200}	$Area_{200}$	DF	V_{OC}	J_{SC}	FF
**CCL_{lam}**	1.000									
**$Area_{lam}$**	0.976	1.000								
**CCL_{pi}**	0.958	0.915	1.000							
**$Area_{pi}$**	0.990	0.979	0.909	1.000						
**CCL_{200}**	0.219	0.099	0.482	0.084	1.000					

**Area$_{200}$**	0.592	0.404	0.655	0.537	0.588	1.000				
**DF**	0.793	0.776	0.594	0.863	0.329	0.440	1.000			
***V*oc**	0.283	0.435	0.034	0.400	0.835	0.444	0.609	1.000		
***J*sc**	0.897	0.900	0.733	0.949	0.213	0.426	0.973	0.601	1.000	
**FF**	0.884	0.912	0.715	0.940	0.262	0.326	0.952	0.670	0.993	1.000
**α**	0.958	0.874	0.934	0.939	0.327	0.794	0.782	0.090	0.846	0.800
**s**	0.853	0.758	0.956	0.776	0.690	0.797	0.435	0.253	0.559	0.516
**1/τ_1**	0.126	0.037	0.404	0.012	0.983	0.430	0.457	0.820	0.320	0.351
**1/τ_2**	0.027	0.132	0.279	0.100	0.951	0.633	0.398	0.950	0.347	0.420
**μ_{hole}**	0.906	0.806	0.841	0.906	0.205	0.817	0.852	0.134	0.869	0.812
**$\mu_{electron}$**	0.902	0.816	0.811	0.916	0.107	0.766	0.900	0.226	0.907	0.855
**HTE**	0.159	0.320	0.090	0.279	0.884	0.543	0.515	0.992	0.496	0.570
**ΔE_1**	0.070	0.098	0.312	0.054	0.944	0.684	0.339	0.937	0.295	0.372
**ΔE_2**	0.653	0.474	0.685	0.612	0.504	0.993	0.542	0.336	0.523	0.427
**ΔE_3**	0.206	0.390	0.005	0.308	0.779	0.598	0.439	0.970	0.470	0.560

	**α**	**s**	**1/τ_1**	**1/τ_2**	**μ_h**	**μ_e**	**HTE**	**ΔE_1**	**ΔE_2**	**ΔE_3**
**CCL$_{lam}$**										
**Area$_{lam}$**										
**CCL$_{pi}$**										
**Area$_{pi}$**										
**CCL$_{200}$**										
**Area$_{200}$**										
**DF**										
***V*oc**										
***J*sc**										
**FF**										
**α**	1.000									
**s**	0.899	1.000								
**1/τ_1**	0.196	0.601	1.000							
**1/τ_2**	0.206	0.542	0.912	1.000						
**μ_h**	0.979	0.808	0.053	0.136	1.000					
**μ_e**	0.963	0.754	0.044	0.038	0.995	1.000				
**HTE**	0.038	0.373	0.855	0.981	0.012	0.106	1.000			
**ΔE_1**	0.258	0.573	0.893	0.998	0.196	0.099	0.974	1.000		
**ΔE_2**	0.842	0.798	0.339	0.539	0.874	0.833	0.440	0.594	1.000	
**ΔE_3**	0.032	0.298	0.722	0.935	0.028	0.057	0.975	0.937	0.504	1.000

'DF' is short for driving force, and 'HTE' for hole transfer efficiency. Person correlation coefficients represent the correlation of two parameters, thus only half of the table is filled with values.

Supplementary Table 11. Photovoltaic parameters based on acceptors NITI and ITIC under illumination of AM 1.5G, 100 mW cm^{-2}.

Condition	V_{OC} (V)	J_{SC} (mA·cm^{-2})	FF (%)	PCE (%)
PM6:NITI [a]	0.890<br>(0.890±0.001)	17.31<br>(17.21±0.26)	55.41<br>(54.71±0.82)	8.53<br>(8.35±0.37)
PM6:PM7:NITI	0.911<br>(0.910±0.001)	17.87<br>(17.69±0.31)	54.82<br>(54.48±0.55)	8.85<br>(8.61±0.24)
PM6:PM7:NITI:PCBM	0.916<br>(0.916±0.001)	18.13<br>(18.00±0.29)	55.67<br>(55.00±0.81)	9.24<br>(9.02±0.32)
PM7:NITI	0.924<br>(0.924±0.001)	14.05<br>(13.52±0.71)	55.59<br>(54.67±1.03)	7.22<br>(6.94±0.46)
PM6:ITIC [b]	1.003<br>(1.003±0.001)	13.99<br>(13.54±0.49)	62.6<br>(62.01±0.74)	8.78<br>(8.44±0.31)
PM6:PM7:ITIC	1.012<br>(1.012±0.001)	14.12<br>(13.98±0.37)	62.4<br>(61.76±0.57)	8.92<br>(8.89±0.19)
PM6:PM7:ITIC:PCBM	1.009<br>(1.009±0.001)	14.21<br>(13.91±0.24)	64.1<br>(63.89±0.66)	9.19<br>(9.01±0.24)
PM7:ITIC	1.006<br>(1.006±0.001)	14.10<br>(13.77±0.41)	58.5<br>(56.97±1.33)	8.30<br>(7.92±0.58)

[a]The device architecture is ITO/PEDOT:PSS/active layer/PFNDI-Br/Ag; D:A=1:1, D concentration = 10 mg ml^{-1} in CB with 1.5% 1-CN, following with 85°C TA treatment. [b]The device architecture is ITO/PEDOT:PSS/active layer/PFNDI-Br/Ag; D:A=1:1, D concentration = 8 mg ml^{-1} in CF with 0.5% DIO, following with 100°C TA treatment.

Supplementary Figures

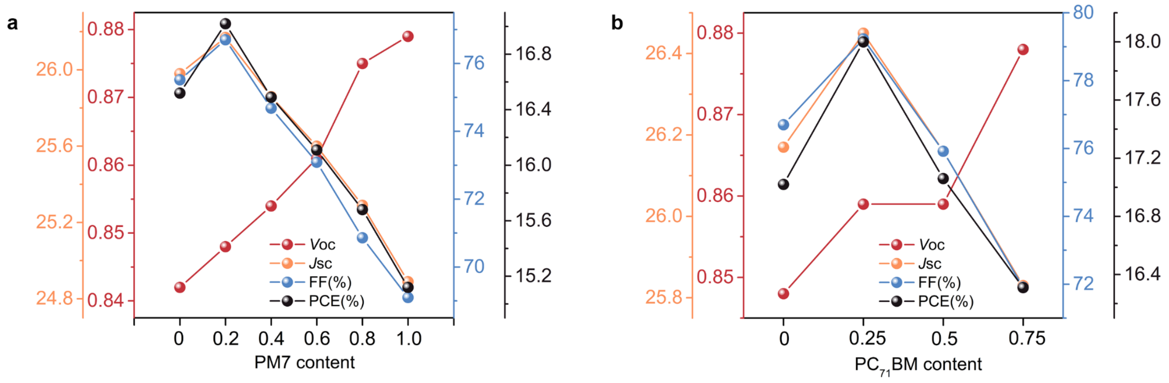

Supplementary Fig. 1. Detailed photovoltaic parameters of devices with different **a,** PM7 and **b,** PC$_{71}$BM contents.

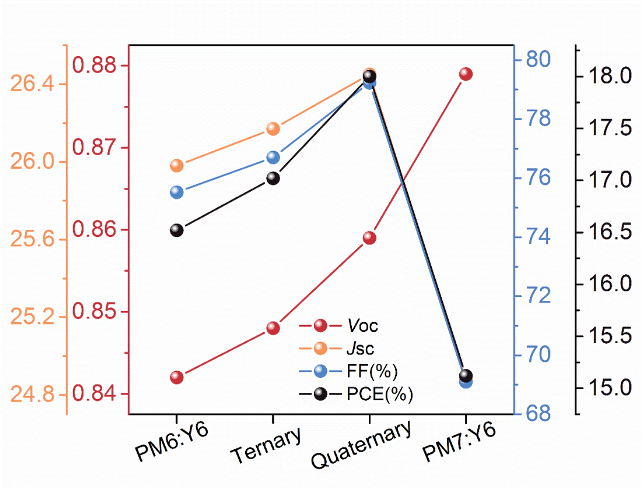

Supplementary Fig. 2. Detailed photovoltaic parameters of devices with different components.

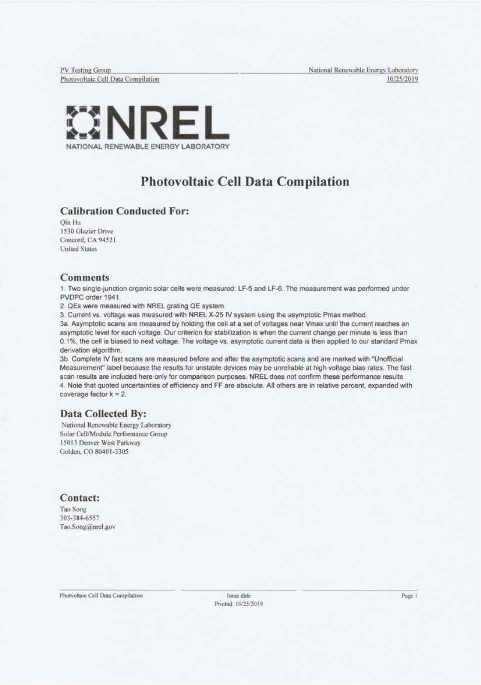

PV Testing Group
Photovoltaic Cell Data Compilation

National Renewable Energy Laboratory
10/25/2019

NREL
NATIONAL RENEWABLE ENERGY LABORATORY

Photovoltaic Cell Data Compilation

Calibration Conducted For:
Qin Hu
1530 Glazier Drive
Concord, CA 94521
United States

Comments
1. Two single-junction organic solar cells were measured: LF-5 and LF-6. The measurement was performed under PVDPC order 1941.
2. QEs were measured with NREL grating QE system.
3. Current vs. voltage was measured with NREL X-25 IV system using the asymptotic Pmax method.
3a. Asymptotic scans are measured by holding the cell at a set of voltages near Vmax until the current reaches an asymptotic level for each voltage. Our criterion for stabilization is when the current change per minute is less than 0.1%, the cell is biased to next voltage. The voltage vs. asymptotic current data is then applied to our standard Pmax derivation algorithm.
3b. Complete IV fast scans are measured before and after the asymptotic scans and are marked with "Unofficial Measurement" label because the results for unstable devices may be unreliable at high voltage bias rates. The fast scan results are included here only for comparison purposes. NREL does not confirm these performance results.
4. Note that quoted uncertainties of efficiency and FF are absolute. All others are in relative percent, expanded with coverage factor k = 2.

Data Collected By:
National Renewable Energy Laboratory
Solar Cell/Module Performance Group
15013 Denver West Parkway
Golden, CO 80401-3305

Contact:
Tao Song
303-384-6557
Tao.Song@nrel.gov

Photvoltaic Cell Data Compilation — Issue date, Printed: 10/25/2019 — Page 1

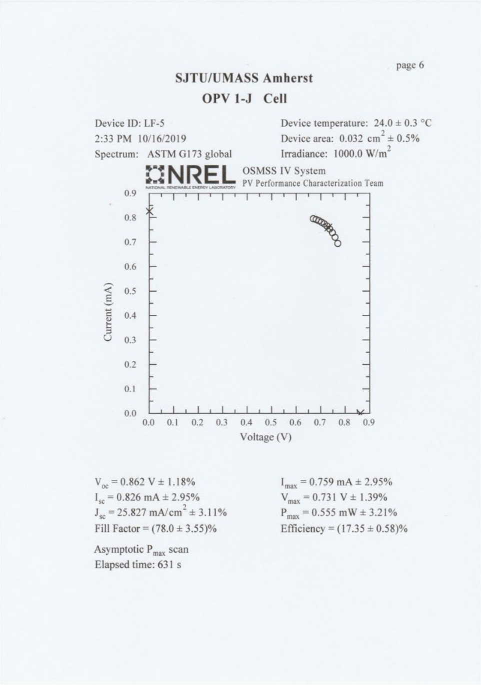

page 6

SJTU/UMASS Amherst
OPV 1-J Cell

Device ID: LF-5
2:33 PM 10/16/2019
Spectrum: ASTM G173 global

Device temperature: 24.0 ± 0.3 °C
Device area: 0.032 cm^2 ± 0.5%
Irradiance: 1000.0 W/m^2

NREL OSMSS IV System
PV Performance Characterization Team

V_{oc} = 0.862 V ± 1.18%
I_{sc} = 0.826 mA ± 2.95%
J_{sc} = 25.827 mA/cm^2 ± 3.11%
Fill Factor = (78.0 ± 3.55)%

I_{max} = 0.759 mA ± 2.95%
V_{max} = 0.731 V ± 1.39%
P_{max} = 0.555 mW ± 3.21%
Efficiency = (17.35 ± 0.58)%

Asymptotic P_{max} scan
Elapsed time: 631 s

Supplementary Fig. 3. Original pages of the OSC certificate results by the National Renewable Energy Laboratory (NREL) in the U.S.A. The device is measured with a mask of 0.032cm^2, giving a PCE of 17.35%.

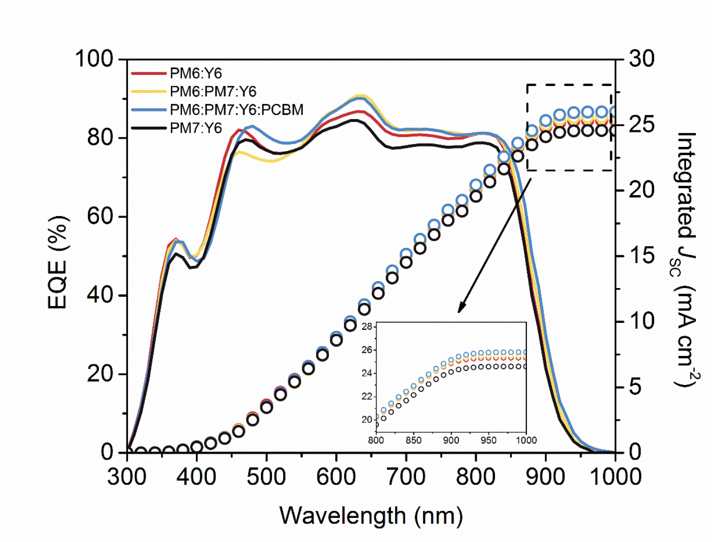

Supplementary Fig. 4. External quantum efficiency (EQE) profiles of different blended devices and integrated J_{SC} (25.34 mA cm^{-2}, 25.61 mA cm^{-2}, 26.01 mA cm^{-2}, 24.59 mA cm^{-2}, respectively).

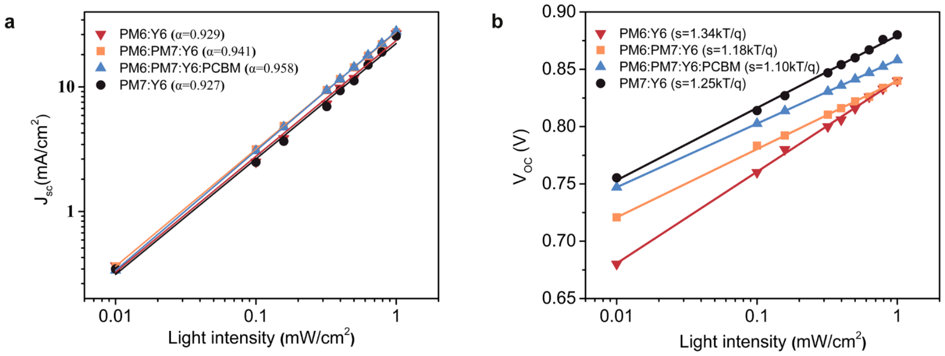

Supplementary Fig. 5. Recombination analysis. a, Characteristics of J_{SC} versus light intensity. **b,** V_{OC} versus light intensity for binary, ternary and quaternary devices.

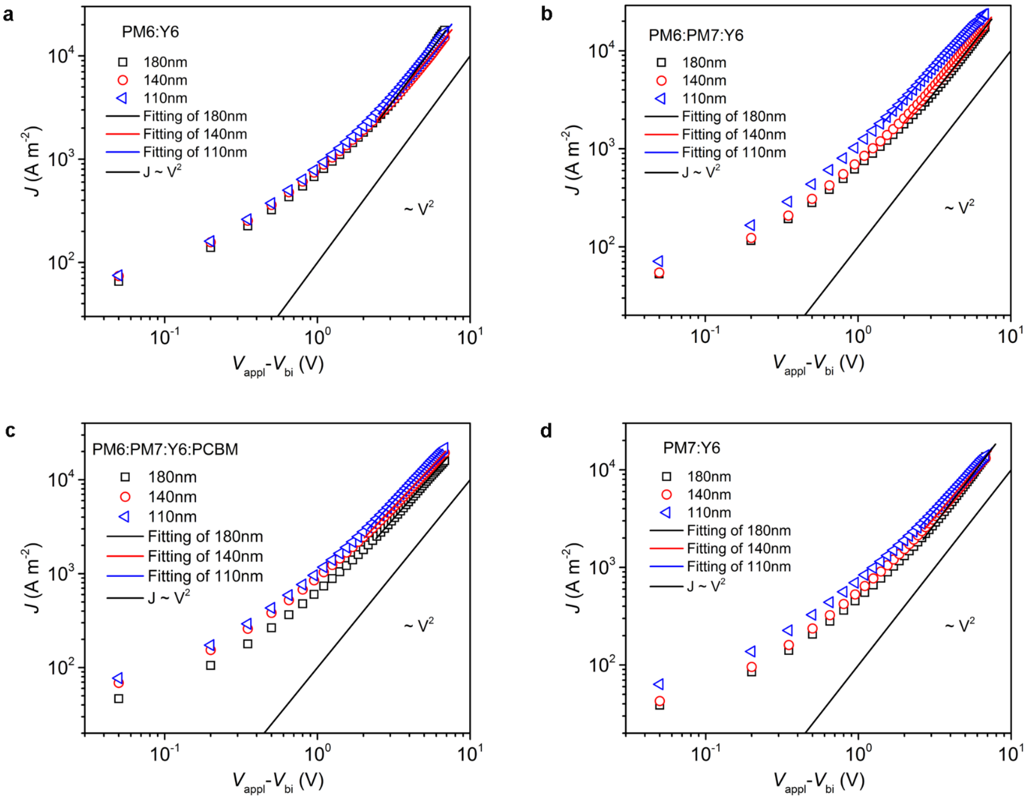

Supplementary Fig. 6. Electron mobility measurement. SCLC measurement of electron-only devices of different thickness with black symbols for 180nm, red symbols for 140nm, and blue symbols for 110nm. The lines are the fitting results according to Mott-Gurney law.

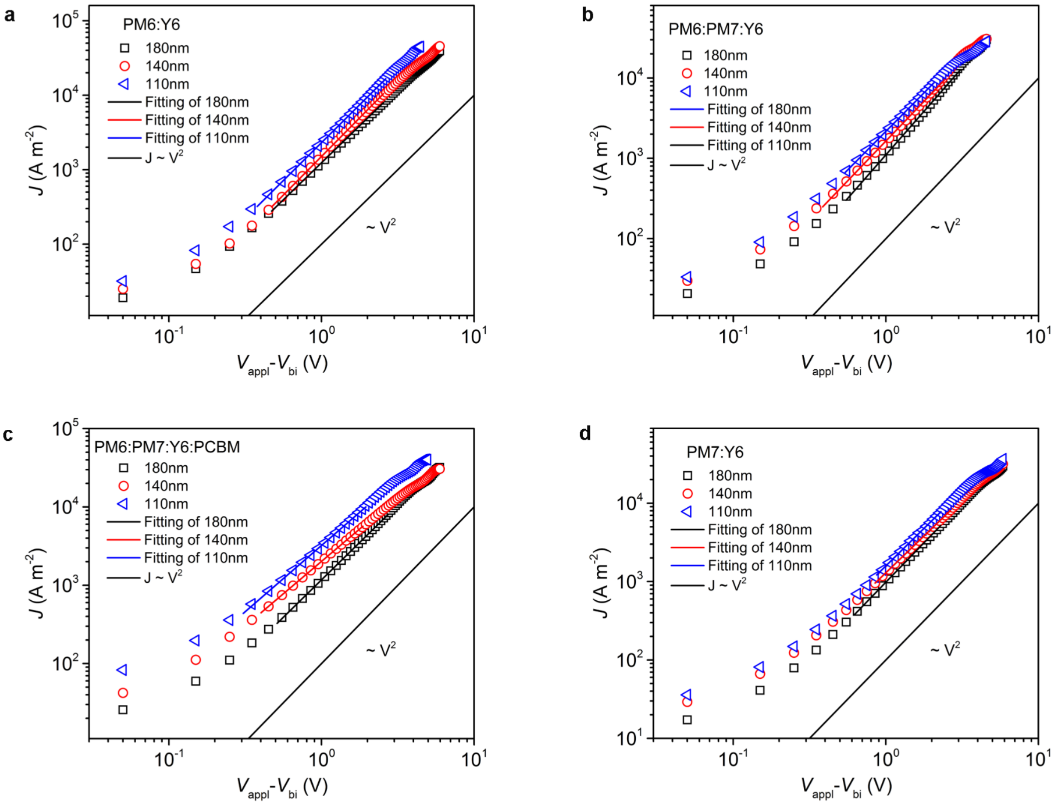

Supplementary Fig. 7. Hole mobility measurement. SCLC measurement of hole-only devices of different thickness with black symbols for 180nm, red symbols for 140nm, and blue symbols for 110nm. The lines are the fitting results according to Mott-Gurney law.

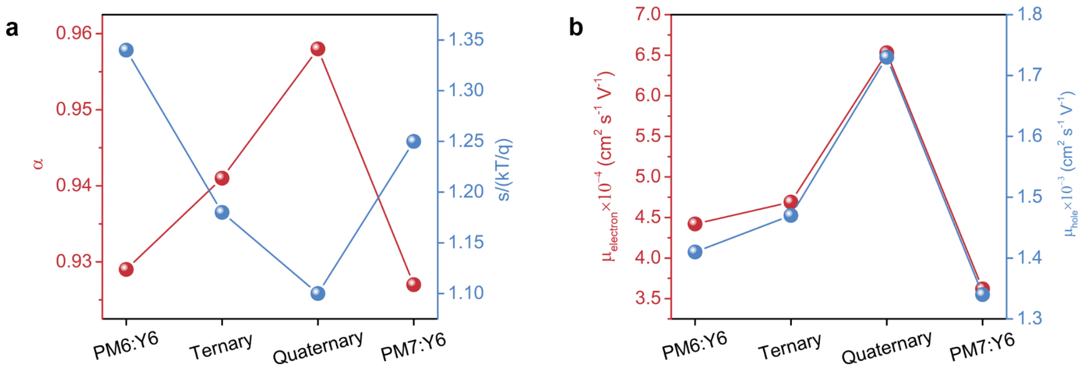

Supplementary Fig. 8 a, α (red) and nkT/q (blue) values based on J_{SC} and V_{OC} dependence on light intensity of devices. **b,** Electron (red) and hole mobilities (blue) under different processing conditions.

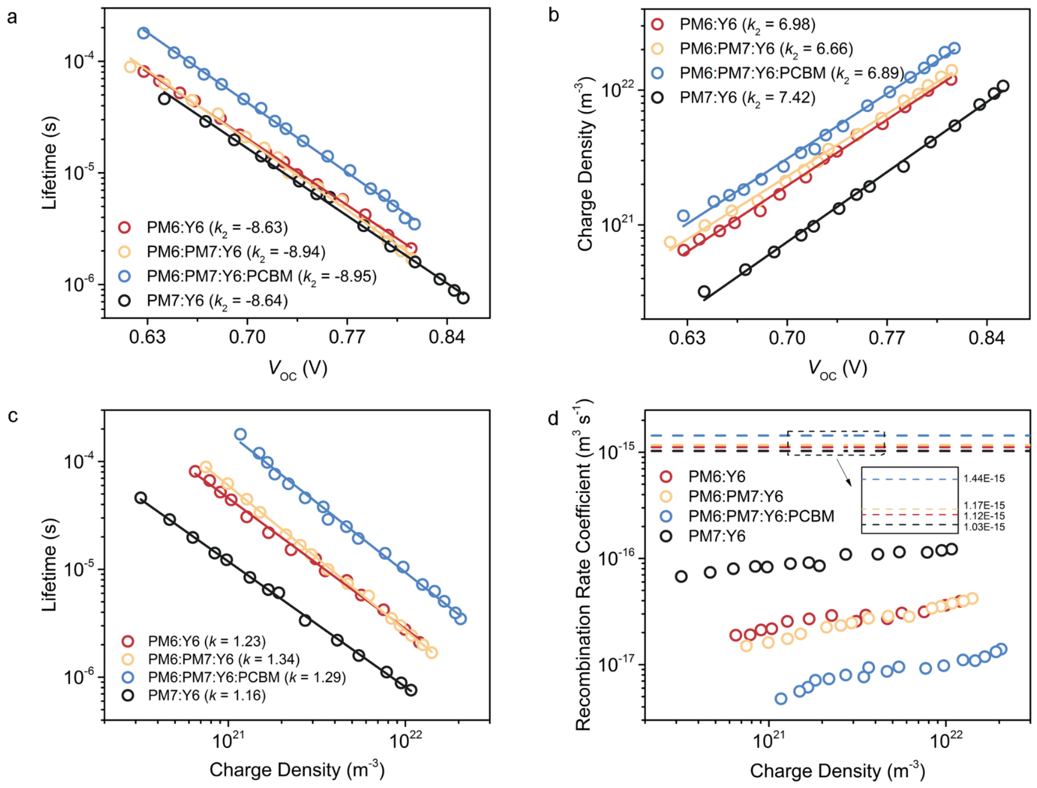

Supplementary Fig. 9. a, Lifetime and **b**, density of charge-carriers under different V_{OC} conditions. **c**, Charge lifetime in the devices as a function of charge density. The solid lines represent a best fit to power law dependence. **d**, Measured nongeminate recombination rate coefficient for devices. The dash lines represent Langevin recombination rate coefficient for different devices.

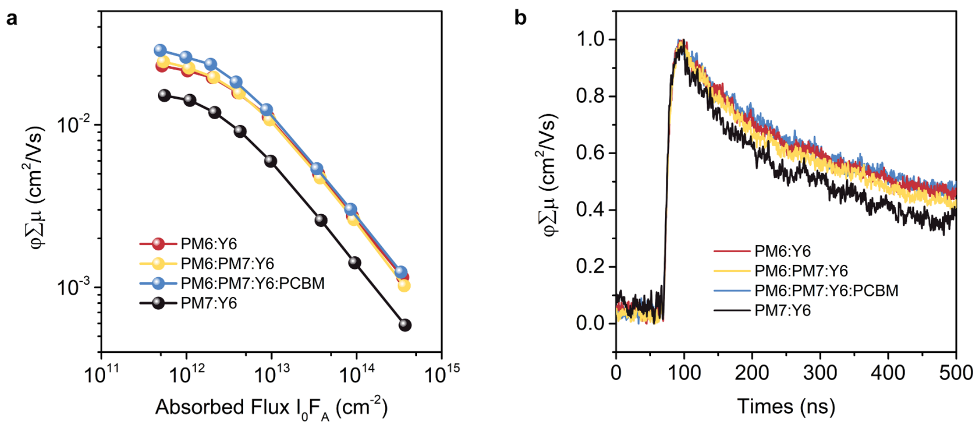

Supplementary Fig. 10. a, Time-resolved microwave conductivity (TRMC) measurement and **b,** normalized photoconductivity transient spectra of different blended films.

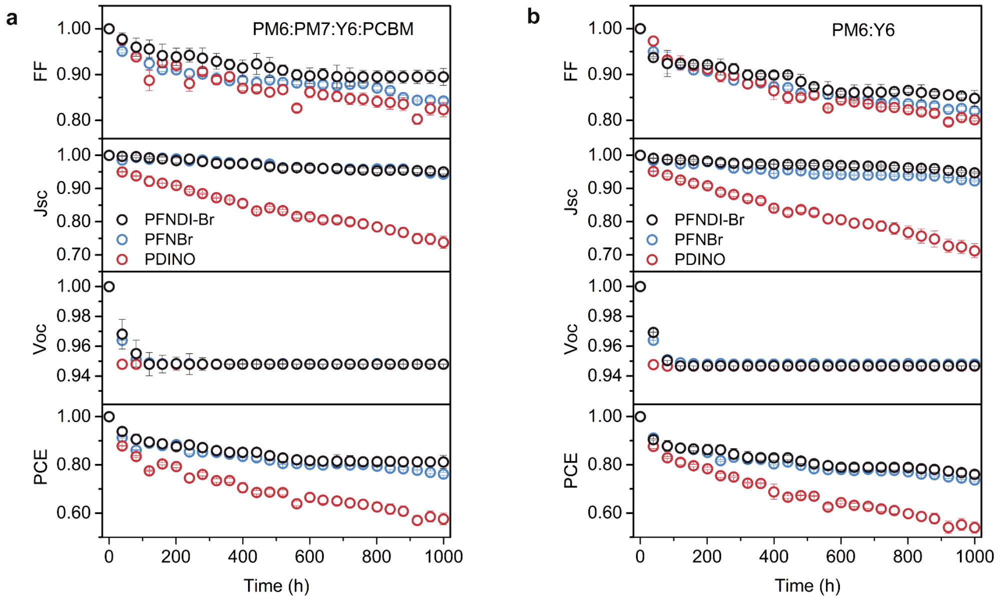

Supplementary Fig. 11. Normalized PCE, V_{OC}, J_{SC} and FF plotted against aging time for **a,** PM6:PM7:Y6:PC_{71}BM and **b,** PM6:Y6 with different electron transport layers (black circle for PFNDI-Br, blue circle for PFN-Br and red circle for PDINO) under illumination equivalent to ~ 1 sun for 1000h (20 devices statistics).

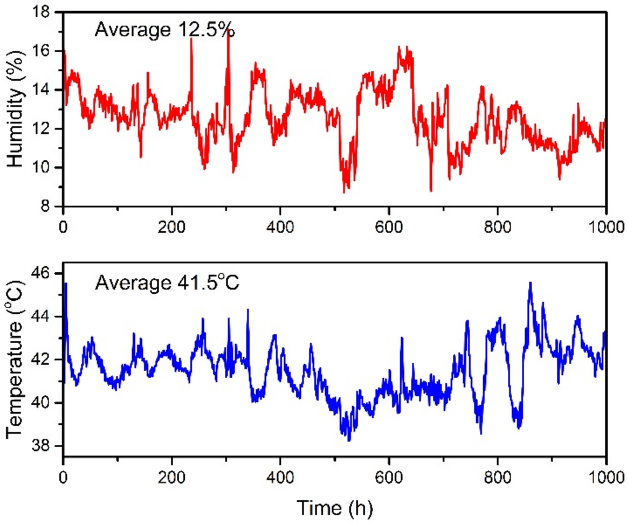

Supplementary Fig. 12. Temperature and humidity during the stability test under continuous illumination.

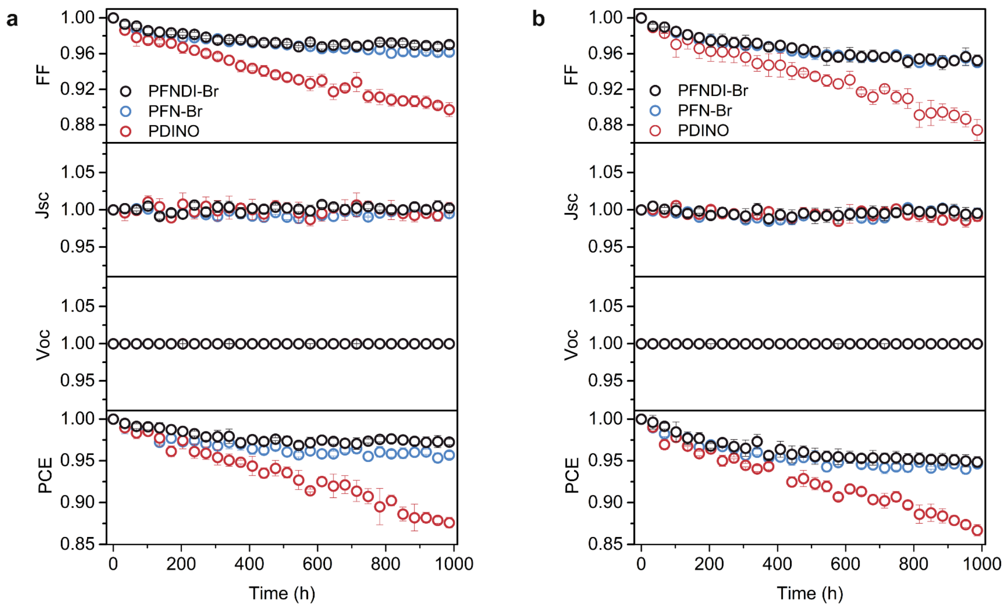

Supplementary Fig. 13. Normalized PCE, V_{OC}, J_{SC} and FF plotted against aging time for **a,** PM6:PM7:Y6:PC_{71}BM and **b,** PM6:Y6 with different electron transport layers (black circle for PFNDI-Br, blue circle for PFN-Br and red circle for PDINO) stored in dark space for 1000h (20 devices statistics).

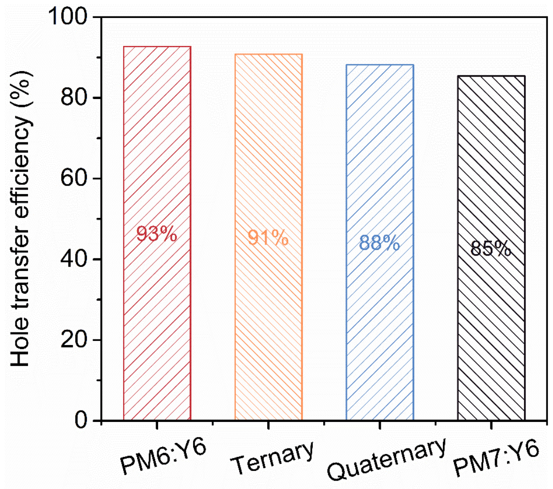

Supplementary Fig. 14. The hole transfer efficiency in different blended films.

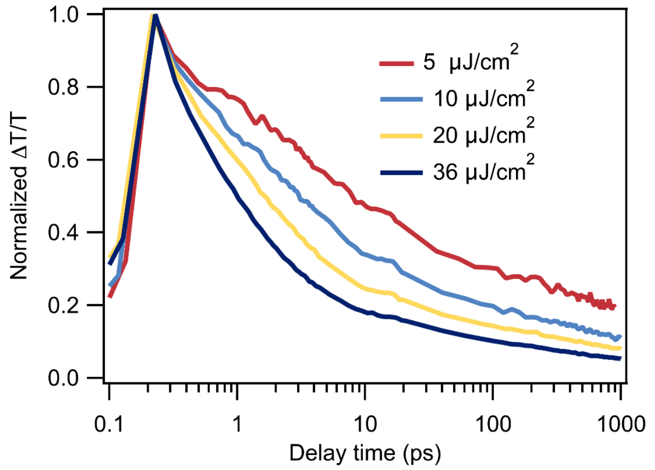

Supplementary Fig. 15. Polaron bleach dynamics of quaternary blend films at different fluences.

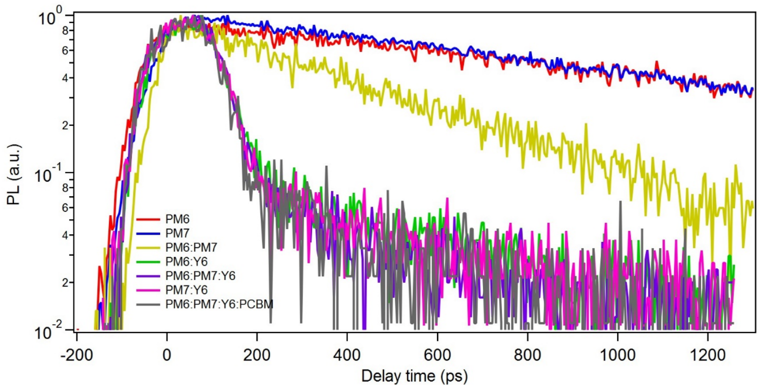

Supplementary Fig. 16. Time-Resolved Photoluminescence (TRPL) measurements of different films.

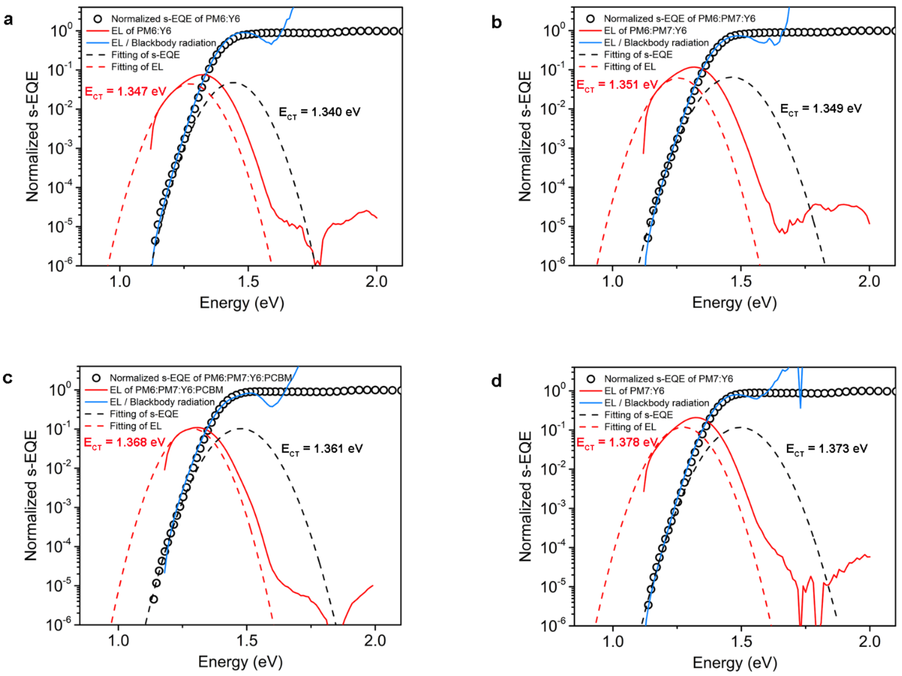

Supplementary Fig. 17. The directly measured quantum efficiency (s-EQE, black circle), electroluminescence spectrum (red line) and corresponding Gaussian Fitting curve (black and red dash line) for **a,** PM6:Y6, **b,** PM6:PM7:Y6, **c.** PM6:PM7:Y6:PCBM and **d.** PM7:Y6. The blue line is the external quantum efficiency determined by ϕ_{EL}/ϕ_{bb}, where ϕ_{bb} is the blackbody radiation.

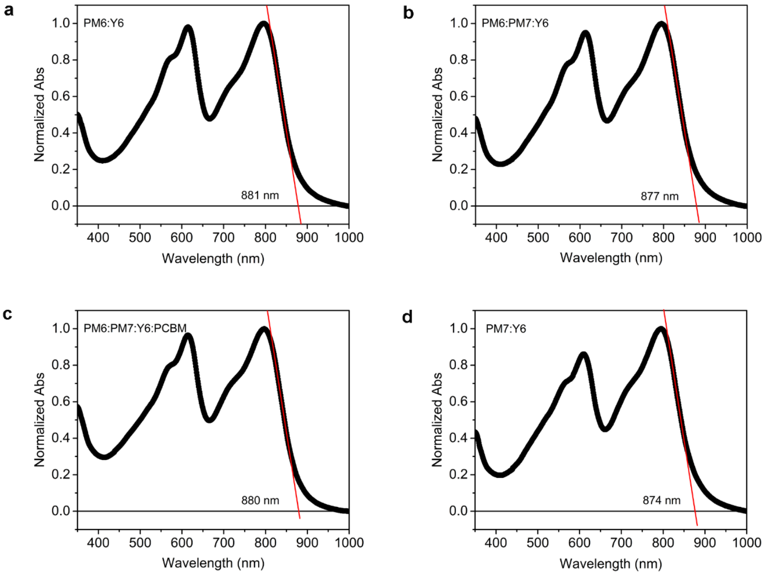

Supplementary Fig. 18. The extracting of E_g by the onset of absorption.

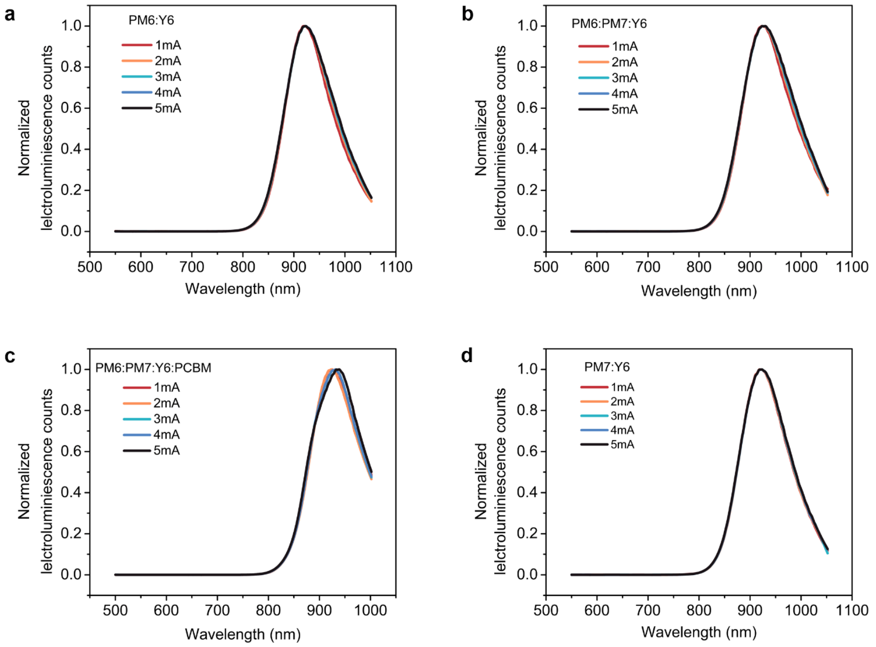

Supplementary Fig. 19. Electroluminescence spectra of devices based on the blended films with different applied currents.

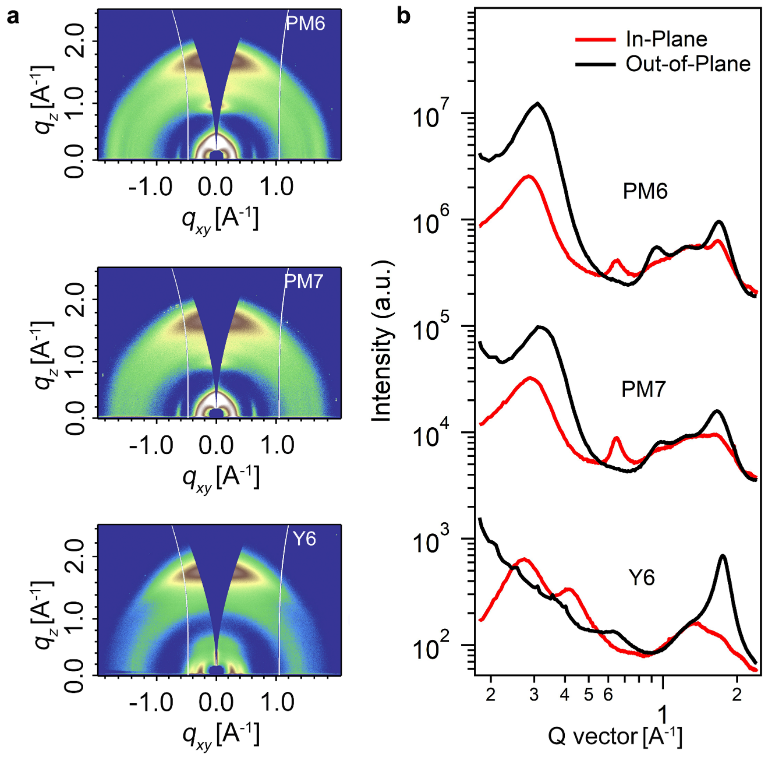

Supplementary Fig. 20. Morphology of single-component thin film. a, 2D GIXD patterns of the pure film. **b,** Out-of-plane (black) and in-plane (red) line cut profiles of the 2D GIXD data.

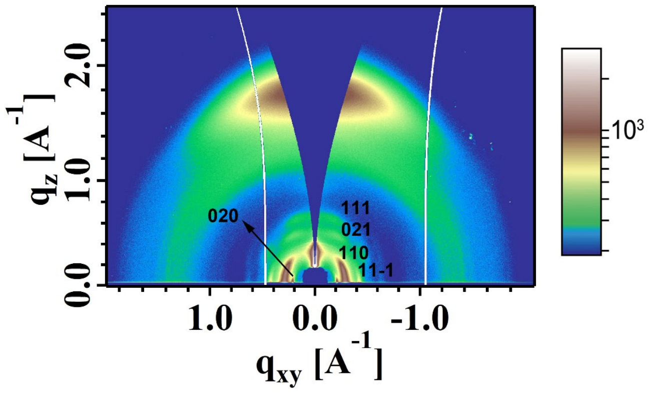

Supplementary Fig. 21. Miller index calibration of Y6 crystals.

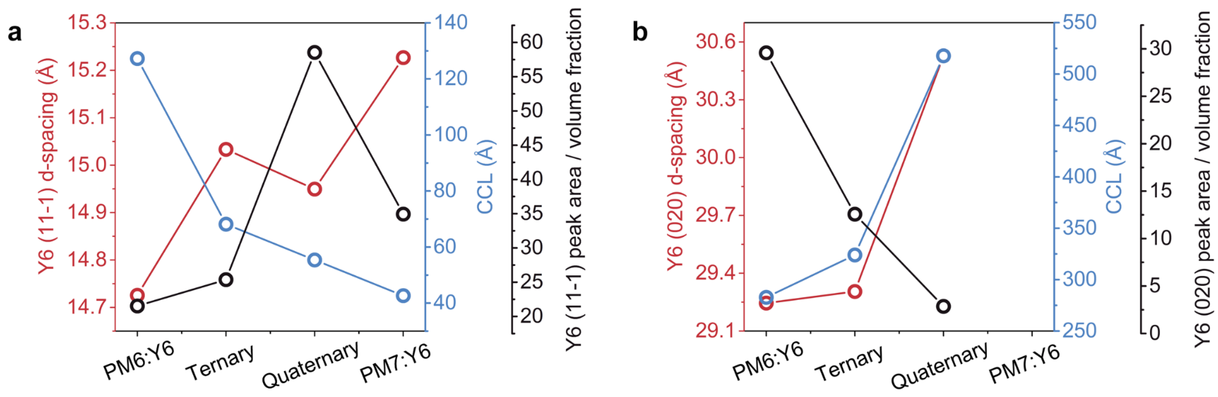

Supplementary Fig. 22. D-spacing, CCL and peak area/volume fraction of **a,** Y6 (11-1) and **b,** Y6 (020) diffraction peaks for blended films with different composition. It should be noted that Y6 (020) peak disappears in PM7:Y6 blend and could not be fitted.

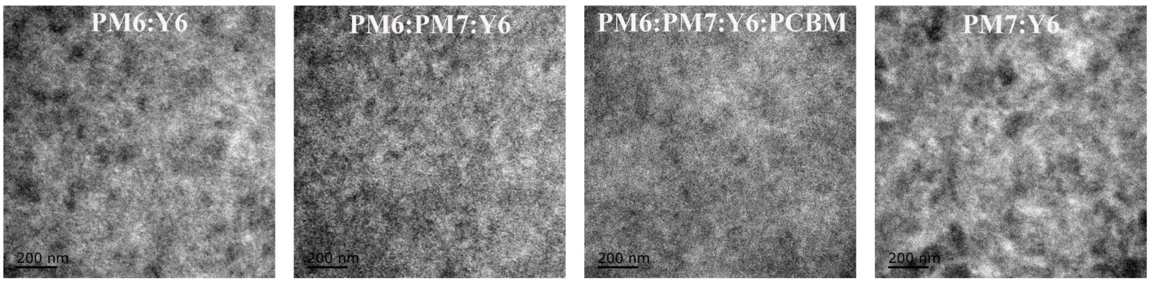

Supplementary Fig. 23. Transmission electron microscopy (TEM) of different blended films.

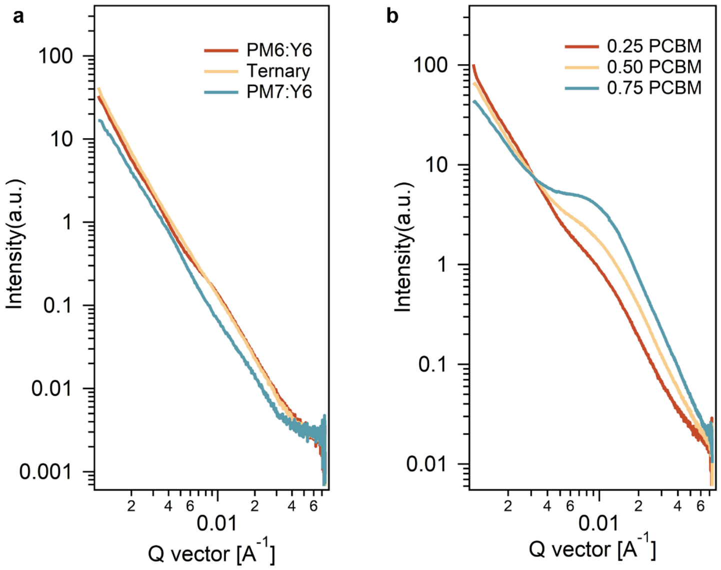

Supplementary Fig. 24. RSoXS profiles of **a,** binary, ternary blends and **b,** quaternary blends with different $PC_{71}BM$ contents.

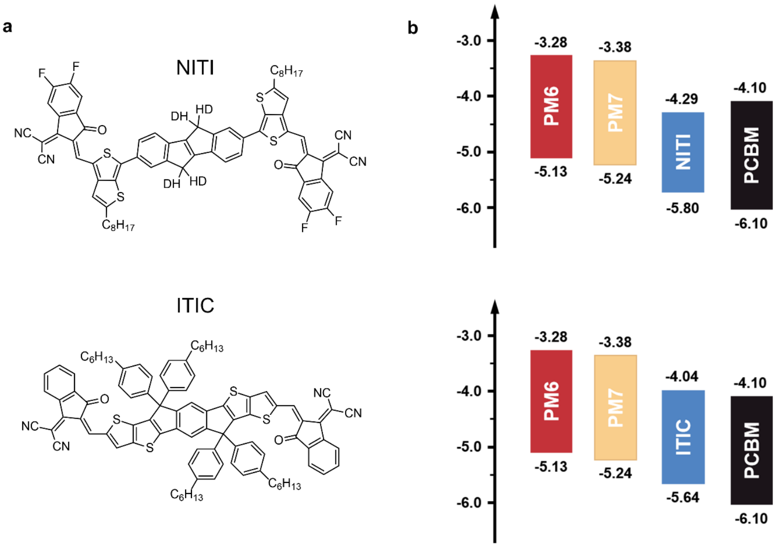

Supplementary Fig. 25. a, Chemical structures, and **b,** energy level alignment of the quaternary blends with NITI and ITIC.

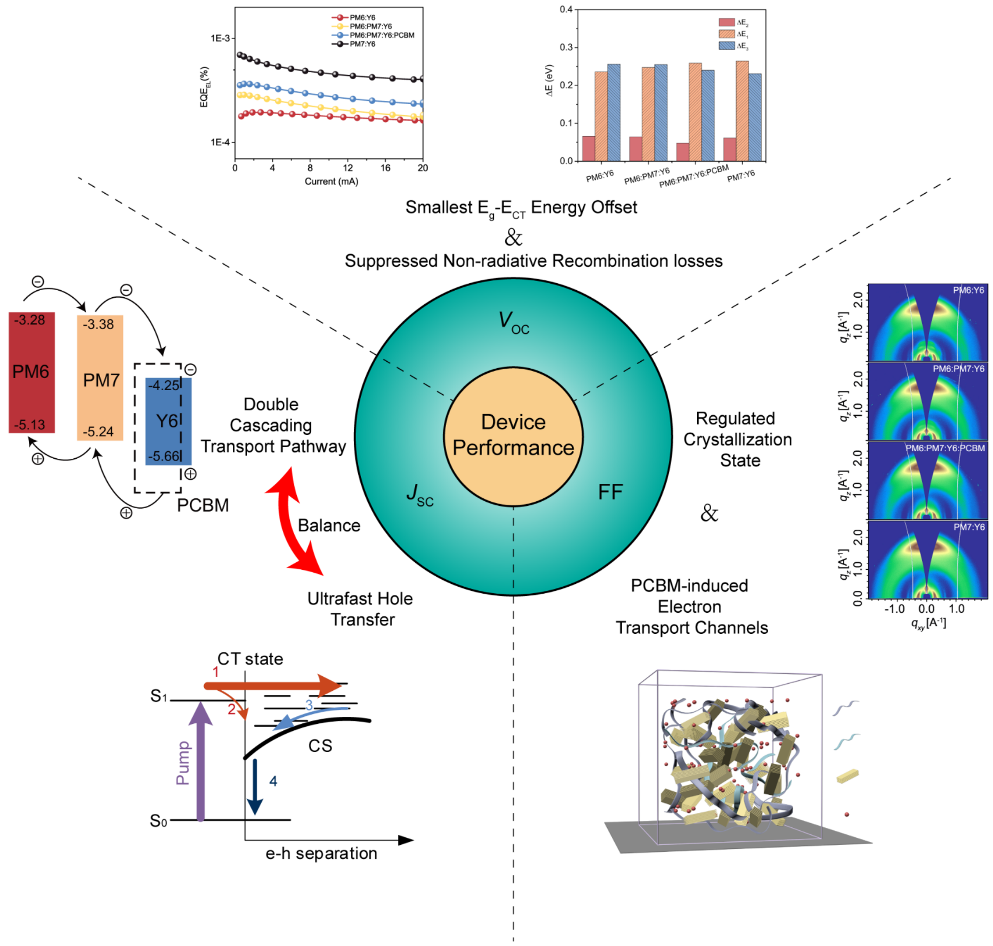

Supplementary Fig. 26. Summary of the quaternary blends construction and how to achieve all-round improvement of device performance.
