# Supplementary material for: Single-layered organic photovoltaics with double cascading charge transport pathways: 18% efficiencies
Source: Nat Commun. 2021 Jan 12;12:309. doi: 10.1038/s41467-020-20580-8 (PMC7803987; doi:10.1038/s41467-020-20580-8)
Supplement: Supplementary file 3 — Solar Cells Reporting Summary [file 41467_2020_20580_MOESM3_ESM.pdf]

nature research

Corresponding author(s): Feng Liu

Solar Cells Reporting Summary

Nature Research wishes to improve the reproducibility of the work that we publish. This form is intended for publication with all accepted papers reporting the characterization of photovoltaic devices and provides structure for consistency and transparency in reporting. Some list items might not apply to an individual manuscript, but all fields must be completed for clarity.

For further information on Nature Research policies, including our data availability policy, see Authors & Referees.

Experimental design

Please check: are the following details reported in the manuscript?

1. Dimensions

Item	Yes/No	Details
Area of the tested solar cells	☒ Yes ☐ No	Section "Device Fabrication"
Method used to determine the device area	☐ Yes ☒ No	The area of our device area was determined and certificated at National Renewable Energy Laboratory (NREL). However, we do not have the detailed information of the method.

2. Current-voltage characterization

Item	Yes/No	Details
Current density-voltage (J-V) plots in both forward and backward direction	☒ Yes ☐ No	The current-voltage (J-V) curve in forward is supplied in Figure. 1e. Both forward and backward scans were conducted, which yielded identical results.
Voltage scan conditions<br>*For instance: scan direction, speed, dwell times*	☒ Yes ☐ No	Section "Device Characterization"
Test environment<br>*For instance: characterization temperature, in air or in glove box*	☒ Yes ☐ No	Section "Device Characterization"
Protocol for preconditioning of the device before its characterization	☐ Yes ☒ No	No preconditioning protocol.
Stability of the J-V characteristic<br>*Verified with time evolution of the maximum power point or with the photocurrent at maximum power point; see ref. 7 for details.*	☒ Yes ☐ No	The stability under storage and operating condition were provided, as shown in Fig. 1g, Supplementary Fig. 11 and Supplementary Fig. 13.

3. Hysteresis or any other unusual behaviour

Item	Yes/No	Details
Description of the unusual behaviour observed during the characterization	☐ Yes ☒ No	No hysteresis or other unusual behaviour was observed during the characterization of the solar cells. In general, organic solar cells do not have hystereisis problems.
Related experimental data	☐ Yes ☒ No	No hysteresis or other unusual behaviour was observed during the characterization of the solar cells.

4. Efficiency

Item	Yes/No	Details
External quantum efficiency (EQE) or incident photons to current efficiency (IPCE)	☒ Yes ☐ No	As shown in Supplementary Fig. 4
A comparison between the integrated response under the standard reference spectrum and the response measure under the simulator	☒ Yes ☐ No	As shown in Supplementary Fig. 4
For tandem solar cells, the bias illumination and bias voltage used for each subcell	☐ Yes ☒ No	Our devices are all single junction.

5. Calibration

Item	Yes/No	Details
Light source and reference cell or sensor used for the characterization	☒ Yes ☐ No	Section "Device Characterization", DM-40S3, SAN-EI ELECTRIC, Japan
Confirmation that the reference cell was calibrated and certified	☒ Yes ☐ No	Section "Device Characterization", Oriel PN 91150V, Newport, USA., NREL calibrated

Calculation of spectral mismatch between the reference cell and the devices under test

☐ Yes ☒ No

The spectral mismatch factor was determined at National Renewable Energy Laboratory (NREL). We do not have the detailed information for the method.

6. Mask/aperture

Size of the mask/aperture used during testing

☒ Yes ☐ No

The mask area is calibrated by NREL, which can be seen in supporting information

Variation of the measured short-circuit current density with the mask/aperture area

☐ Yes ☒ No

We only measured performances with masks.

7. Performance certification

Identity of the independent certification laboratory that confirmed the photovoltaic performance

☒ Yes ☐ No

The performance was certified by National Renewable Energy Laborotary

A copy of any certificate(s)
Provide in Supplementary Information

☒ Yes ☐ No

Supplementary Fig. 3

8. Statistics

Number of solar cells tested

☒ Yes ☐ No

The device performance statistics was based on 40 devices

Statistical analysis of the device performance

☒ Yes ☐ No

results were included in Fig. 1f

9. Long-term stability analysis

Type of analysis, bias conditions and environmental conditions
For instance: illumination type, temperature, atmosphere humidity, encapsulation method, preconditioning temperature

☒ Yes ☐ No

Section "Device Characterization", Fig. 1g and Supplementary Fig. 11-13 for details
